# First observations of core-transiting seismic phases on Mars

**DOI:** 10.1073/pnas.2217090120

**Published:** 2023-04-24

**Authors:** Jessica C. E. Irving, Vedran Lekić, Cecilia Durán, Mélanie Drilleau, Doyeon Kim, Attilio Rivoldini, Amir Khan, Henri Samuel, Daniele Antonangeli, William Bruce Banerdt, Caroline Beghein, Ebru Bozdağ, Savas Ceylan, Constantinos Charalambous, John Clinton, Paul Davis, Raphaël Garcia, Domenico Giardini, Anna Catherine Horleston, Quancheng Huang, Kenneth J. Hurst, Taichi Kawamura, Scott D. King, Martin Knapmeyer, Jiaqi Li, Philippe Lognonné, Ross Maguire, Mark P. Panning, Ana-Catalina Plesa, Martin Schimmel, Nicholas C. Schmerr, Simon C. Stähler, Eleonore Stutzmann, Zongbo Xu

**Affiliations:** ^a^School of Earth Sciences, University of Bristol, Bristol BS8 1RJ, United Kingdom; ^b^Department of Geology, University of Maryland, College Park 20742; ^c^Institute of Geophysics, ETH Zurich, Zurich 8092, Switzerland; ^d^Institut Supérieur de l’Aéronautique et de l’Espace ISAE-SUPAERO, Toulouse 31055, France; ^e^Royal Observatory of Belgium, Brussels 1180, Belgium; ^f^Institute of Geochemistry and Petrology, ETH Zurich, Zurich 8092, Switzerland; ^g^Université Paris Cité, Institut de physique du globe de Paris, CNRS, Paris 75005, France; ^h^Sorbonne Université, Muséum National d’Histoire Naturelle, UMR CNRS 7590, Institut de Minéralogie, de Physique des Matériaux et de Cosmochimie, Paris 75005, France; ^i^Jet Propulsion Laboratory, California Institute of Technology, Pasadena, CA 91109; ^j^Department of Earth, Planetary, and Space Sciences, University of California, Los Angeles, CA 90095; ^k^Department of Applied Mathematics and Statistics & Department of Geophysics, Colorado School of Mines, Golden, CO 80401; ^l^Department of Geophysics, Colorado School of Mines, Golden, CO 80401; ^m^Department of Electrical and Electronic Engineering, Imperial College London, London SW7 2AZ, United Kingdom; ^n^Swiss Seismological Service, ETH Zurich, Zurich 8092, Switzerland; ^o^Department of Geosciences, Virginia Tech, Blacksburg, VA 24061; ^p^DLR, Institute of Planetary Research, Berlin 12489, Germany; ^q^Department of Geology, University of Illinois Urbana-Champaign, Urbana, IL 61801; ^r^Geosciences Barcelona - CSIC, Barcelona 08028, Spain; ^s^Physik-Institut, Universität Zürich, Zurich 8057, Switzerland

**Keywords:** Mars, core evolution, planetary structure

## Abstract

We present the first observations of seismic waves propagating through the core of Mars. These observations, made using seismic data collected by the InSight geophysical mission, have allowed us to construct the first seismically constrained models for the elastic properties of Mars’ core. We observe core-transiting seismic phase SKS from two farside seismic events detected on Mars and measure the travel times of SKS relative to mantle traversing body waves. SKS travels through the core as a compressional wave, providing information about bulk modulus and density. We perform probabilistic inversions using the core-sensitive relative travel times together with gross geophysical data and travel times from other, more proximal, seismic events to seek the equation of state parameters that best describe the liquid iron-alloy core. Our inversions provide constraints on the velocities in Mars’ core and are used to develop the first seismically based estimates of its composition. We show that models informed by our SKS data favor a somewhat smaller (median core radius = 1,780 to 1,810 km) and denser (core density = 6.2 to 6.3 g/cm^3^) core compared to previous estimates, with a P-wave velocity of 4.9 to 5.0 km/s at the core–mantle boundary, with the composition and structure of the mantle as a dominant source of uncertainty. We infer from our models that Mars’ core contains a median of 20 to 22 wt% light alloying elements when we consider sulfur, oxygen, carbon, and hydrogen. These data can be used to inform models of planetary accretion, composition, and evolution.

## Significance

Mars has a liquid iron alloy core at its center. Using seismic data gathered by the InSight mission, we have made the first observations of seismic waves traveling through Mars’ core. We use the travel times of core-transiting seismic waves, relative to ones which remain in the mantle, to constrain properties of the core and construct the first models of the elastic properties of the entire planet. Our results are consistent with a core rich in sulfur, with smaller fractions of oxygen, carbon and hydrogen.

Recent results from the InSight geophysical mission to Mars (1) have revealed the layered nature of the red planet, illuminating its crustal structure (2–5), mantle velocities (6–9), and a mid-mantle seismic discontinuity associated with the phase transition of olivine (10). InSight has also recorded reflections from a large impedance contrast that is interpreted as the core–mantle boundary (CMB) (11). The Martian core is mostly, if not totally, liquid—the planet’s tidal response can be used to rule out an entirely solid core (12–19). Formed early in Mars’ history (20, 21), the core is an iron alloy rich in light elements with sulfur proposed as the main light element present based on Martian meteorite geochemistry e.g., refs. 22 and 23. In contrast to the Earth and Mercury, no global magnetic field is currently generated by the Martian core, though crustal magnetism (24, 25) suggests the presence of a magnetic field early in the planet’s history, pointing toward a possible evolution of Mars’ core over the planet’s lifetime, e.g., refs. 26, 27 and 28. Beyond these core facts, a great deal about Mars’ central interior remains unknown, including the elastic properties and composition of its core (29).

Initial seismic observations using core-reflected seismic waves (ScS) reported a core radius of 1, 830 ± 40 km (11), at the upper bound of prelanding estimates e.g., refs. 13–16, 18, 30–34. These results are compatible with the independent findings of InSight’s Rotation and Interior Structure Experiment (RISE) (35), which has measured the effect of the liquid core on the nutation of Mars (36). The inferred core radius and simultaneous estimation of a relatively low core density have motivated questions about its composition: If only sulfur is considered as an alloying element, an implausibly high core sulfur fraction is required to match the core density whilst satisfying constraints on mass, moment of inertia, and tidal response of the planet (11). Though the observation of seismic waves reflected from Mars’ CMB has helped constrain the core radius, and geophysical and cosmochemical inversions have sought to infer its average density and composition e.g., ref. 37, observations of seismic waves that directly probe core properties have been lacking to date.

Seismological investigations of core-transiting waves have been made on Earth for more than a century (38, 39), where both seismometers and hypocenters of large earthquakes are distributed around the globe. Analyses of their travel times have constrained the seismic properties of the liquid outer core e.g., refs. 40 and 41, supporting the presence of light elements (42) and enabling the estimation of its equation of state e.g., ref. 43. With just a single broadband seismometer and seismic sources smaller than those routinely detected on the Earth, comparable observations have proved more challenging on Mars. Here, we present and analyze the implications of new observations, which constitute the first detection of seismic waves transiting the Martian core.

## Observations and Analysis

### The Events.

**Fig. 1.**
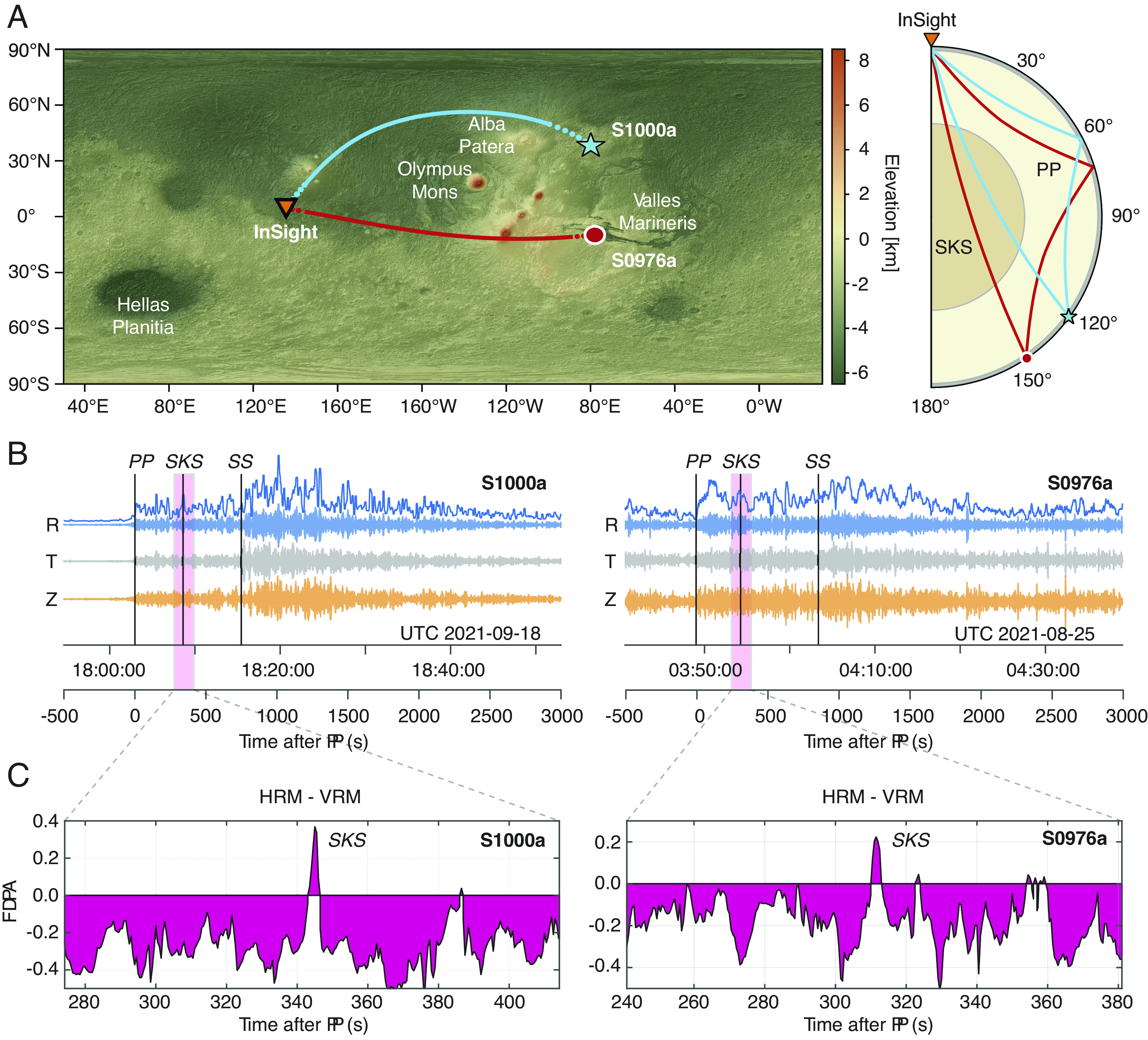
Location map, seismic data, and frequency-dependent polarization analysis for events S0976a and S1000a. (*A*) Locations of the two farside events, S0976a (red circle) and S1000a (blue star), and the InSight seismometer (orange triangle). The dotted lines show the SKS path in the mantle, and the solid lines depict the part of the SKS path in Mars’ core. [Surface topography model from ref. 57. Raypaths of seismic phases SKS and PP are shown in the same colors as events. SKS travels through the core; PP remains in the mantle. PP may have multiple arrivals at this epicentral distance (10); we show the path of the first propagating wave. SS, used together with PP as a reference phase, has a very similar path to PP (*SI Appendix*, Figs. S15 and S16). (*B*) Radial (blue), transverse (gray), and vertical (orange) component seismograms for S1000a (*Left*) and S0976a (*Right*), together with travel time picks. Above the radial component, we show its envelope. (*C*) Horizontal-vertical summed FDPA intensity as a function of time (analysis method A). The strong horizontally polarized signal is interpreted as the arrival of SKS.

The InSight mission deployed a very broadband seismometer (44) onto the surface of Mars in late 2018, leading to the identification of numerous seismic events (45–48). To date, only two seismic events have been identified as located on the opposite hemisphere of Mars to the InSight lander (49). These events (Fig. 1*A*) are designated S0976a and S1000a, corresponding to the first seismic events detected on Sols (InSight Martian mission days) 976 and 1,000, respectively.

These events were located using phases identified as PP and SS—waves which travel down to a depth of nearly 1200 km in the Martian mantle and reflect from the surface of Mars mid-way along their path. The Mars Quake Service (MQS) estimated the epicentral distances of S0976a and S1000a to be 146^°^ ± 7^°^ and 128^°^ ± 19^°^, respectively, with Mars-calibrated magnitudes (50) *M*_*w*_^*Ma*^ of 4.2 and 4.1, respectively (47, 49). Uncertainty on marsquake locations is considerable because they depend on seismic models of Mars’ layered crust and mantle and, for source depth determination, the unequivocal identification of depth phases e.g., refs. 7 and 8. Fortunately, orbital imaging combined with data from InSight revealed S1000a to be an impact at a distance of 125.9^°^ and a backazimuth of ∼34^°^ (51), though the exact time of the impact is not known. We are therefore able to precisely locate the source of S1000a, and fix its depth to zero, removing this source of uncertainty from our work.

### Phase Detection.

At the distances corresponding to S0976a and S1000a, SKS, which travels as a shear wave in the crust and mantle and as a compressional wave in the core (Fig. 1*A*), will be the first core-transiting phase to arrive (*SI Appendix*, Fig. S11). Core phase PKP, which travels its entire path as a compressional wave, is not predicted at these distances, as the expected velocities of the core and mantle require PKP arrivals to be confined to areas close to the antipode of a marsquake, unlike the wider detection zone on Earth.

SKS arrives between the PP and SS signals (Fig. 1*B*) and with an amplitude comparable to that of PP on the radial component seismograms (*SI Appendix*, Figs. S15 and S16). ‘Radial’ is defined as the horizontal direction along the great circle path from the event to the station and is perpendicular to the ‘transverse’ (horizontal) component and the vertical component. At the distances corresponding to S0976a and S1000a, SKS has key distinctive attributes that we exploit to facilitate its detection: SKS should be a vertically polarized shear wave (SV) with little horizontal shear (SH) motion on the transverse component (assuming seismic anisotropy is weak); due to its steep incidence angle, SV energy of SKS should be linearly polarized and strongest on the radial component of motion; SKS is expected to have a waveform related to the shape of SS (assuming minimal complexity introduced at the SS bounce point) through a Hilbert transform; SKS arrives in a time window where few interfering phases should be present (travel time curves are shown in *SI Appendix*, Fig. S11) so that arrivals detected with the correct characteristics can be assigned to be SKS with reasonable confidence. Radial anisotropy, which has been detected in the Martian crust (52–54), should not impact these attributes of SKS unless there is an as-yet undiscovered more complex anisotropic texture present.

Detection of SKS, and measurement of its travel time relative to either PP or SS, is made using several different methods. These can be broken into two categories: i) arrival detection techniques, which seek to detect wavepackets of energy in the seismic record with the correct characteristics, and ii) correlation-based methods, which find the time delay between different packets of energy associated with seismic arrivals. Three arrival-based methods (methods A–C) and two correlation-based methods (methods D and E) were applied to the data.

We present here results from frequency-dependent polarization analysis (FDPA), an arrival detection technique which assesses whether signals are present at a range of frequencies and have the required polarization. This method (labeled as method A in this work) has previously been implemented for InSight data and successfully identified the core-reflected ScS phases (11) as well as the minor-arc surface waves on Mars (5). Fig. 1 depicts the seismic data together with the excess of total horizontally polarized energy as compared to vertically polarized energy as a function of time since the MQS PP arrival time. Glitches (i.e., nonseismic spikes in the recorded data) can complicate the interpretation of seismic phases on InSight data (55, 56) and have been removed (*SI Appendix*, section S2.8).

For event S1000a, the SKS signal is identified using the FDPA method (Fig. 1*C*) as the horizontally polarized energy arriving ∼340 s after PP (method A in Table 1). This is the most coherent signal in the relevant time window.

**Table 1.** SKS differential travel times measured using five different methods, three arrival detection methods, and two cross-correlation methods (*SI Appendix* for details)

	S0976a			S1000a		

Method	SS-PP (s)	SKS-PP (s)	SS-SKS (s)	SS-PP (s)	SKS-PP (s)	SS-SKS (s)
A (arrival det.)	858.5	311.0	547.5	–	344.2	401.4
B (arrival det.)	853.4	298.3	555.1	752.3	334.8	417.5
C (arrival det.)	859.0	310.0^*^	549.0^*^	745.6	339.0	406.6
D (cross-corr.)	855.0	309.0	546.0	–	–	–
E (cross-corr.)	846.0	297.4	548.6	–	–	–
Average	854.4	303.9	549.2	749.0	339.3	408.5
SD	5.2	7.1	4.1	4.7	4.7	8.2

Times marked ^*^ are insufficiently confident for use in inversions.

In addition to method A, measurements of SKS differential travel times were also sought for S1000a using the four other complementary methods, B–E (Table 1 and *SI Appendix*, section S2), with the two alternate arrival detection methods (B & C) able to detect SKS. The three different arrival detection methods (A–C) enhance slightly different characteristics of the waveform, with different optimal frequency bands, and therefore find slightly different differential arrival times. The two cross-correlation methods (D & E) were unable to measure SKS differential travel times for S1000a, possibly due to the more emergent nature of the SS signal for this event (49). We note that the PP and SS arrivals determined using method C were employed to get the SKS differential travel times reported for *Method A*. *SI Appendix*, section S2.1 summarizes and compares the five different methods employed.

The SKS signal from S0976a is identified using the FDPA method (method A in Table 1) as the horizontally polarized energy arriving ∼310 s after PP (Fig. 1*C*). In this case, the two cross-correlation–based methods (D & E) also found signals identified as SKS, and one further waveform identification method (B) found an SKS arrival. The final waveform identification technique (method C) did not find a single observation clearly identifiable as SKS, we include their estimate here for completeness. The SKS-PP measurements have a range of 14 s whilst the SS-SKS times have a range of 9 s. The use of the cross-correlation methods provides further justification of our identification of SKS. Methods D and E use the SS waveform as a template to correlate with SKS, so that we know the two wavepackets have related shapes, as we would expect for waves generated by the same marsquake.

Environmental noise, particularly that due to wind, can be prominent on InSight’s seismic records (46, 48, 58–60). We verify, using the method of ref. 60, that the signals identified as SKS for both events are not associated with excess environmental energy (*SI Appendix*, section S2.7). In light of this analysis and the expected amplitudes of SKS (*SI Appendix*, section S3.4), we conclude that our SKS detections and associated travel times are robust. In this investigation, it has proven vital to have 3-component recordings to ensure the phase identified was indeed SKS; future missions to other planetary bodies e.g., ref. 61 will benefit if 3-component seismometers can be employed in preference to vertical-component instruments alone.

The average differential travel times of all reliable observations are used in our inversion, with an assigned uncertainty of 10 s. This assigned uncertainty is greater than the SD of the travel time picks, in order to account for other sources of uncertainty. For example, potential three-dimensional (3D) velocity variations in the mantle can also affect differential travel times (18). We quantify these using ray theoretical calculations through candidate thermochemical models of Mars’ interior as in ref. 62 and find that they are comparable to the assigned observational uncertainty.

We can assess, using forward modeling (63), how uncertainties in the event depth and epicentral distance would affect SKS differential travel times. The source depth for S0976a is not well constrained, as no clear depth phases (or surface waves) are identified. A standard MQS event depth of 50 km is assigned to this event; changing the source depth by 30 km changes the differential travel times by less than 5 s (*SI Appendix*, Fig. S12) as the arrival times of both SKS and the mantle reference phase are affected in the same way by changing the event depth. Effects are more substantial when the epicentral distance is altered—changing the epicentral distance by 5^°^ modifies PP-SKS time by around 10 s, but around 30 s for SS-SKS differential travel times. Thus, the uncertainty in the locations of S0976a makes it challenging to accurately assess the seismic velocity inside Mars’ core. The known impact location obviates this concern for S1000a.

Shear wave phase SKKS, which is reflected from the underside of the CMB, and Sdiff, which diffracts along the CMB (*SI Appendix*, Figs. S15 and S16), are not observed for either event; using current models (11), we anticipate that they would both arrive approximately 100 s after SKS, with radial and transverse polarizations, respectively. The similar travel times of these two phases are due to the very small velocity difference expected between compressional wavespeed in Mars’ uppermost core and shear wavespeed in Mars’ lowermost mantle, so that traveling across the uppermost core or along the CMB takes similar amounts of time. The absence of identifiable arrivals permits some possible inferences to be made about the lowermost mantle. SKKS travels to and from the CMB with a more oblique angle than SKS; if the lowermost mantle contains any partial melt e.g., ref. 64, SKKS would be more attenuated than SKS. The absence of Sdiff may be caused by a similar phenomenon. Alternatively, Sdiff is observed at relatively long periods on Earth e.g., ref. 65, which would make it difficult to detect given the range of frequencies typically usable on Mars. While Pdiff has been detected (9, 49, 51), it remains the case that Sdiff has not yet been observed in marsquake waveforms.

## Core Properties

We first compare our observations to predictions from the InSight_KKS21_GP model (3, 6, 11). This model was created with Bayesian inversions using a geophysical parameterization (6, 11) and has been adapted to have a three-layer, 48 km thick crust, consistent with (3, 6, 7, 56). We note i) that the P wave velocities in this model are not constrained by seismic data below a depth of 800 km (6), which means that predictions of PP arrival times might be less reliable than SS; and ii) the core velocity structure in this model is not seismically constrained. The observations for S0976a are close to predictions for the MQS epicentral distance (within 10 s for both PP-SKS and SKS-SS for an event depth of 50 km). The same model predicts times for S1000a, known to be an impact at a distance of 125.9^°^, which are within 20 s for SKS-PP and 15 s for SS-SKS. The PP-SS time of S1000a is within two seconds of the prediction made using InSight_KKS21_GP, suggesting that the discrepancy between our observed and predicted SKS times may be in part due to the lack of previous seismic constraints on the elastic properties of Mars’ core.

While a core sulfur content close to the eutectic makes the formation of an inner core in Mars unlikely at temperatures above ∼1200 to 1500 K (29, 66), the detection of SKS signals from S0976a and S1000a places a bound on the maximum size of any Martian inner core. As SKS reaches a minimum radius of ∼750 km, we expect that an inner core, if one exists, would be smaller than this radius. Further constraints on the presence or absence of an inner core are expected from InSight’s RISE instrument (35).

With two events and two reference phases, there are four differential travel times that must be satisfied by a reasonable seismic model of Mars’ interior. Relying on the crust and mantle of InSight_KKS21_GP and systematically changing the velocity at the CMB and core velocity gradient, we can seek the properties of the core that best fit the data. Such an analysis (*SI Appendix*, Fig. S14) suggests that a range of core properties is broadly compatible with the observations. However, the core velocity and gradient of InSight_KKS21_GP do not make predictions which fall close (within one SD) to the average SKS differential travel times. Instead, this forward modeling suggests that the seismic velocity of the core may be higher than that of the model, while the velocity gradient might be slightly lower. It would be possible to take an existing model of mantle properties and simply seek the core properties which best fit the SKS differential times. However, such an analysis would not allow for the possibility that InSight_KKS21_GP does not represent the velocities of the crust and mantle accurately. Moreover, it would not take into account the uncertainty in the depth and epicentral distance of S0976a. Finally, such an approach would preclude a joint analysis of the correlated uncertainties in mantle and core structure across different mantle-inversion strategies. A joined-up approach to the modeling of Mars’ interior allows us to avoid inadvertently compensating for imperfect models of the mantle by selecting incorrect properties for the core—trade-offs can be more easily visualized and understood. Thus, we opt to invert our data for new models of Mars’ interior.

### Seismic Inversion.

Exploiting core-transiting phases, we can refine estimates of the internal properties of Mars. Indeed, without core-transiting phases, the elastic properties of the core can only be constrained indirectly and generally under the assumption that the core is composed of an iron–sulfur alloy e.g., refs. 14–16, 30. In refs. 7, 11 and 8, the core radius was estimated using the travel times of core-reflected waves together with geophysical data. Here, we invert seismic data including the relative travel times of core-transiting phases, requiring that the resulting models fit the mean planetary density (3.935 ± 0.0012 g/cm^3^) and mean normalized moment of inertia of Mars (0.3634 ± 0.00006) (13).

We conduct three separate sets of Bayesian inversions, two of which use the SKS differential travel times presented here. The first set of inversions— hereafter referred to as producing “*geophysical*” models—use the travel times from direct, reflected, and converted crustal, mantle, and core seismic phases gathered in ref. 7 and have a similar modeling approach for the mantle parameterization. The second and third sets of inversions use the “*geodynamical*” parameterization of (67) with their dataset of direct and reflected mantle- and core-sensitive travel times (8). The third set of inversions differs from the second in that it does not include SKS differential travel times, so that it can serve as a point of reference. Full details of inversion methods are provided in *SI Appendix*, section S4.

All sets of inversions are parameterized assuming that the core’s thermo-elastic properties can be described by an isentropic third-order Birch–Murnaghan equation of state (68). The core’s elastic properties are therefore described by three parameters: density, *ρ*_*CMB*_, the adiabatic bulk modulus, *K*_*CMB*, *S*_, and its pressure derivative, *K*′_*CMB*, *S*_, referenced to CMB conditions. As Mars has no currently active geodynamo, its core may be presently fully conductive, or it may be that the convective region in the core generates insufficient excess entropy to produce a magnetic field (17, 27, 28, 69); therefore, a temperature profile along an isentrope is a first-order assumption, commonly employed in modeling planetary cores. A hotter, isothermal core would change the predicted CMB velocity by only ∼0.5%.

**Fig. 2.**
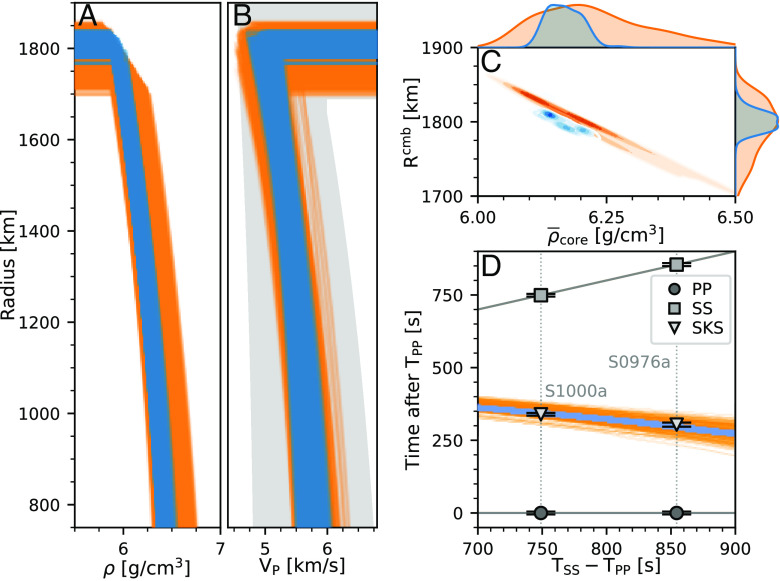
Inversion results for the seismic properties of Mars’ core. *Geophysical* inversion results are shown in blue, *geodynamical* results are shown in orange. (*A* and *B*) Density and seismic velocity models for Mars’ core. In panel (*B*), the gray area indicates the results of *geodynamical* inversions carried out without using the SKS differential travel times. (*C*) Average density and core radius of Mars. The histograms above and to the right display the posterior distributions of the average density and core radius, respectively. (*D*) Observed (black) and predicted (colors) travel times.

The elastic models produced are sieved using the requirement that they fit the SKS differential travel times within twice the uncertainty bounds. This is a conservative choice, intended to account for potential differential travel time variations due to 3D velocity variations within the mantle. It effectively increases the importance of fitting the SKS differential travel times over the mantle phases, which are already the subject of significant investigation (7–9). Inversions were conducted using both the *geophysical* and *geodynamical* parameterizations for the geochemically derived model mantle composition EH45 (23). Compositions described by LF (22), TAY (70), and YMD (71) were also used (*SI Appendix* section S4). Seismic models are shown in Fig. 2 *A* and *B*; models including their crust and mantle components are shown in *SI Appendix*, Fig. S21.

Information on the travel times of core-transiting phases has allowed us to constrain the elastic properties of the core much more directly than before (compare gray and colors in Fig. 2*B*) and, importantly, without making assumptions about the composition of the core and properties of its metallic alloy. Our inversion results show an expected anticorrelation between core radius and density—in order to fit the mass and moment of inertia constraints applied, models with a larger core must have a lower core density. Assuming an EH45 model to inform the mantle composition, we find core radii of 1814 km (interquartile range, IQR: 1,804 to 1,823 km) and 1,799 km (IQR: 1,773 to 1,819 km) for the *geophysical* and *geodynamical* inversions. Assumptions about mantle composition affect these estimates somewhat: the LF and TAY compositions result in a 6 km and 15 km smaller core, while the YMD composition implies the smallest core, with a median radius of 1,779 km. Therefore, while modeling choices lead to different distributions of the core radii (the histogram on the right of Fig. 2*C*), the medians of all distributions fall within ±17 km of each other.

These values can be compared to the first estimates based on ScS travel times (waves reflected from Mars’ CMB) of 1, 830  ±  40 km (11), 1, 840  ±  10 km (37), 1, 845  ±  25 km (7), and 1, 773 ± 41 km when crustal constraints are used in the *geodynamical* inversion of ref. 8. Thus, while mostly consistent within the uncertainties quoted, our results support a slightly smaller core.

When we compare models constructed using EH45 to inform mantle composition, median bulk core densities, *ρ*_*c*_, are 6.21 g/cm^3^ (IQR: 6.18 to 6.24 g/cm^3^) and 6.25 g/cm^3^ (IQR: 6.18 to 6.34 g/cm^3^) for the *geophysical* and *geodynamical* models, respectively. These average core densities depend on assumed mantle composition; for example, in the *geophysical* inversions, replacing the EH45 composition model with the TAY one reduces *ρ*_*c*_ by 0.05 g/cm^3^, while instead using LF increases *ρ*_*c*_ by 0.01 g/cm^3^. The largest median bulk core density is obtained in the *geodynamical* inversions using the YMD mantle composition (*ρ*_*c*_ = 6.35 g/cm^3^, IQR: 6.27 to 6.50 g/cm^3^). Accounting for uncertainties due to assumptions of mantle composition, the range of median core densities we obtain in this study, *ρ*_*c*_ = 6.16 to 6.35 g/cm^3^, is higher than the earlier estimates of 6.0±0.3 g/cm^3^ (11). Within uncertainty, they overlap with estimates obtained by (37) and (7) of 6.15 ± 0.046 g/cm^3^ and 6.1 ± 0.1 g/cm^3^, respectively, and allow us to rule out some of the lighter core scenarios reported in ref. 11.

The P-wave velocity at the top of the core, *V*_*CMB*_, is seismically constrained for the first time by our observation of SKS. We find that, at the CMB, the median seismic P-wave velocity ranges from ∼4.89 to 5.05 km/s, depending on assumptions of mantle composition. Using EH45 to inform the mantle composition, the *geophysical* and *geodynamical* models presented here have median CMB core velocities of 4.94 (IQR: 4.87 to 5.05 km/s) and 4.89 (IQR: 4.78 to 5.01 km/s), respectively. These estimates are lower than those measured for pure liquid iron (e.g., 5.14 ± 0.14 km/s at 20.5 GPa and 2,300 K, 72), providing a further, independent argument in favor of a substantial fraction of light elements in the Martian core. *Geodynamical* inversions using the seismic travel time dataset of ref. 8, but excluding the core-transiting signals, result in a faster median *V*_*CMB*_ of 5.10 km/s and much greater uncertainties (∼110% broader IQR: 4.87 to 5.35 km/s). The extra information contained in the SKS travel times allows us to substantially narrow the estimates of *V*_*CMB*_ relative to premission predictions (15, 16, 18, 30, 32) (Fig. 2). With two events providing SKS observations, there is necessarily some degree of trade-off between the seismic velocity at the CMB and the velocity gradient in the core. Nonetheless, the velocities of our model families are tighter both at the CMB and at depth when we include the SKS data.

The core radius, velocity, and density distributions provided by the *geophysical* and *geodynamical* inversions have different IQRs (Fig. 2). In addition to the distinct inversion priors of the two methods, there are several factors that contribute to these differences: First, the *geodynamical* parameterization (17) relies on a larger number of free parameters than the *geophysical* parameterization (6). Second, the sensitivity of the inverted parameters to the seismic data is different for each method (67, 73). Finally, different choices were made in assembling the travel time datasets used in the two inversion schemes (7, 8).

While our inversions are designed to self-consistently predict velocities at all radii below the CMB—thereby providing estimates of velocity throughout the core—the absence of deeply diving paths reduces our ability to constrain velocity gradients. Thus, because the paths traversed by our SKS observations do not reach the center of Mars (Fig. 1), we display the models only in the top 1,000 km of the core.

By constructing a one-dimensional model of the planet’s elastic properties, we implicitly assume that the portions of crust and mantle of Mars transited by waves from S0976a and S1000a are not too dissimilar to that under InSight. Surface wave analysis indicates that, while crustal velocities are similar north and south of the dichotomy, crustal thicknesses vary substantially (5, 53); this is one of the reasons that we have assigned uncertainties larger than the measurement standard deviations to our differential travel time observations. We have also assumed that there is no distinct molten layer atop Mars’ core–mantle boundary (64). The possible existence of a compositionally distinct molten layer would not only affect estimates of core radius and velocities in the lowermost mantle but also those of temperature and composition at the CMB. As such, joint investigations of the core and lowermost mantle of Mars will be an important future step.

### Interpretations.

Many previous works modeled the core under the approximation of an iron–sulfur alloy, though it is reasonable to assume that Mars’ core contains notable fractions of other light elements e.g., refs. 11, 30, 74 and 37. Inverting for the parameters governing the core equation of state allows us to consistently compute the density and velocity of the core without making recourse to any specific core composition. The resulting core density and velocity can then be compared to that corresponding to liquid iron alloys at Mars’ core pressure and temperature conditions to constrain the nature and abundance of light elements.

We use the seismically derived models presented here to seek out physically consistent core compositions for Mars. Specifically, we identify the combinations of light element abundances in a multicomponent Fe–O–S–C–H alloy, which can produce the P-wave velocity *V*_*CMB*_ and *ρ*_*CMB*_ of the core alloy. Although our seismic inversions also contain information about *K*_*S*_′, this parameter is less well-constrained due to the aforementioned weak constraint on velocity gradient with depth provided by the SKS travel times. Consequently, while a subset of compositional models can reproduce the velocity across the full range of core pressures, we do not limit our discussion to only those core compositions. As for the light element content, we invert for the fraction of S and H in the core, while fixing the amount of O to that of S and mantle FeO following (75), as O is incorporated in the metallic core alongside S during core differentiation. Carbon also is directly related to S, as it is assumed to be at the solubility limit in Fe–S (76).

**Fig. 3.**
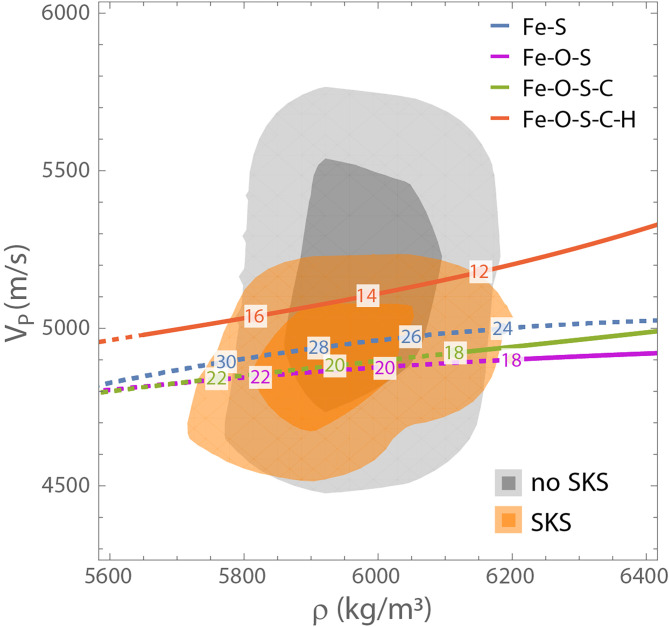
Core velocity and density at the CMB compared with equation of state predictions. Results from the *geodynamical* inversions with and without SKS data are shown, with the lightly and strongly shaded areas indicating 90% and 50% of the models, respectively. The lines correspond to predictions for liquid Fe–S, Fe–O–S, Fe–O–S–C, and Fe–O–S–C–H alloys. Moving along each line corresponds to variations in the amount of sulfur present (wt% S is indicated by the numbers along the line). Lines are dashed where the alloys contain more than 18 wt% sulfur. When present, carbon is at saturation level and hydrogen is fixed at 1 wt%.

We first compare the results of our inversions with equation of state predictions for the velocity and density of different alloys with a variable light element content (Fig. 3). We see that when core properties are not informed by SKS, many of the retrieved models have velocity–density combinations incompatible with core-candidate liquid Fe-alloys, with seismic velocities above those of alloys with > 1wt% hydrogen. The introduction of SKS travel time constraints narrows the permissible velocity–density range for the Martian core, in a way that restricts it to a region that can be accounted for by a number of liquid iron alloys of plausible light element content. Therefore, while not pointing at a specific core composition, the travel times of core-transiting seismic phases provide independent arguments in support of a volatile-rich core, as put forward on the basis of cosmochemical arguments which encompass the chemical and isotopic analysis of Martian meteorites e.g., refs. 22, 23, 34, 70 and 71 and core differentiation models e.g., refs. 30, 74 and 75.

While Fe–S and Fe–O–S alloys could provide a match to the SKS-informed core models, they do so at unreasonably high sulfur fractions (e.g., estimates of reasonable S fractions from 30, 74, are 17 to 19%). Inclusion of C, even at the solubility limit, only marginally reduces the sulfur fraction required. However, adding hydrogen to a Fe–S–O–C alloy increases the core velocity substantially, while requiring much less sulfur to account for the density. The effect of varying CMB temperature and pressure is to broaden these lines, increasing both the range of possible velocities for a given density by ∼100 m/s (*SI Appendix*, Fig. S23) and the width of implied posterior elemental abundance distributions. Finally, we note that a Fe–Si alloy would produce seismically impermissible velocities > 6, 000 m/s (77), independently reinforcing the conclusion, drawn from assessments of the oxidizing conditions of Mars’ formation, that only a negligible amount of Si is present in Mars’ core e.g., ref. 30.

We now turn to the results of our compositional inversions; full details of the methods used are provided in *SI Appendix*, sections S4.4–S4.5. We find that the assumptions made when seeking to match the density and velocity of our core models with compositional models are as important as the seismic inversion choices and, in some cases, can restrict the space of allowed core velocity–density space even more tightly than the SKS observations. When we use EH45 to model the Martian mantle and assume that O, S, C, and H are the light elements in the core, we infer core compositions that contain a median of 16.5 wt% S for the *geodynamical* models and 15.4 wt% S for the *geophysical* models, with IQRs of 15.1 to 18.4 wt% and 14.6 to 16.3 wt%, respectively. The corresponding median core fractions of other light elements are 2.9 to 3.2 wt% O (IQRs span 2.7 to 3.7 wt% for the two families of models), medians of 1.2 to 1.4 wt% C (IQRs span 1.0 to 1.5 wt%) and 0.5 to 0.6 wt% H (IQRs span 0.2 to 0.7 wt%). The S and H contents of the core are, as expected, anticorrelated (*SI Appendix*, Fig. S24)—a core with appropriate density can be achieved with either very little H and a larger amount of S, or a larger fraction of H and a lower amount of S. The choice to model the core as saturated in C has an impact of ∼1 wt% on the quantities of all the other light elements interpreted to be present in the core. The choice of the equation of state for liquid FeH is also critical for these compositional inferences. Thus, we do not stress precise compositional inferences but rather argue that the core-traversing waves presented here are indicative of a core with a high fraction of light elements.

Estimates of the total light element abundance of the Martian core are largely controlled by constraints on core mass, while constraints on core P-wave velocities help to discriminate between different light-element scenarios. Using EH45 to inform the mantle, our compositional inversions find that the Martian core contains a median of 20.3 to 21.4 wt% total light elements (IQRs span 19.5 to 23.4 wt%) for the *geophysical* and *geodynamical* models. This is higher by about ∼1 wt% than the total light element composition one would obtain without SKS travel time data. When considering alternative mantle compositions to EH45 (LF, TAY, or YMD) and resulting mineralogies, median total light element abundances are only affected by < 1 wt%, with the LF composition suggesting the smallest (median of 19.8 wt%) and the YMD composition implying the largest (median of 21.6 wt%) total light element abundances.

**Fig. 4.**
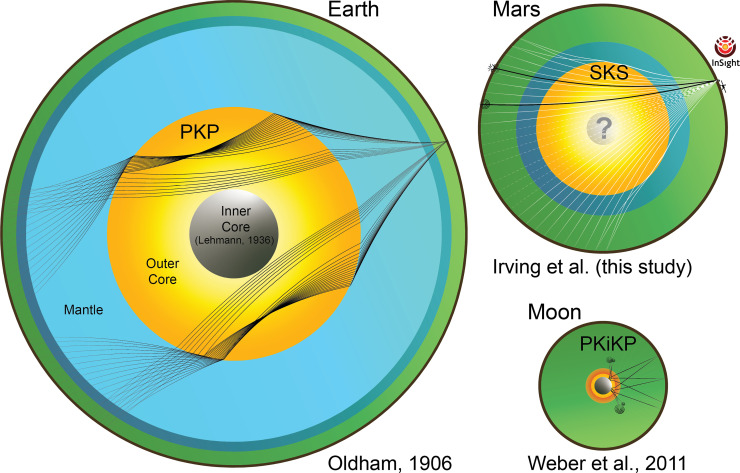
Schematic showing core-transiting ray-paths through the three seismically explored planetary bodies: Earth (38), Mars, and the Moon (78). Earth’s inner core was discovered some thirty years after the outer core (79). Colors within each body correspond to different dominant minerals and phases. Mars has an upper mantle dominated by olivine (shown in green), and a mantle discontinuity corresponding to the post-olivine phase transition (10) indicated by dark blue. On Earth, below the olivine-rich upper mantle and the transition zone, the lower mantle is predominantly bridgmanite (light blue); the lowermost mantle is not shown. The liquid metallic core of each body is shown in shades of yellow, while on the Moon and Earth, the inner core is shown in gray, and the Moon’s partial melt layer is shown in red.

Seismology has long been used to make inferences about the size and composition of Earth’s ∼7,000-km diameter core and permitted the discovery of the much smaller lunar core (Fig. 4). Estimates for the light elements present in the lunar core vary but suggest a negligible fraction of silicon and oxygen and relatively high fractions of sulfur and carbon (29, 80–82). Uncertainties in understanding the lunar core’s composition are substantial in part because core-transiting phases reported by ref. 78 were observed using challenging Apollo-era data. New observations made by the Farside Seismic Suite (83) will shed further light on the lunar core. On Earth, seismic velocities and density have been used to better our understanding of the light elements present in the core. Earth’s liquid outer core is suggested to have only about half the fraction of light elements advocated for Mars’s core, as it is likely to contain less than 2 wt% S, along with no more than 4 wt% Si, less than 6 wt% O and up to 0.25 wt% H e.g., ref. 84. We stress, however, that the limit of 2 wt% S in the Earth’s core is driven by geochemical arguments and volatility trends e.g., refs. 85 and 86, while on the sole basis of thermo-elastic properties, Earth’s outer core could contain up to 10 wt%S (87, 88), or as much as 14.4 wt%S if it is assumed to be the only light element present (84). Consideration of the condensation chemistry of elements in the solar nebula and feeding zones for planetary formation and accretion leads to the expectation that planets formed further from the Sun will contain a larger amount of volatile elements (89, 90). A precise determination of the light element budget of Mars’ core, based on combined geophysical observations, mineral physics, petrological, and cosmochemical constraints will be vital in comparing the processes at play during the formation of the Earth and Mars. Such a comparison could reveal the extent to which differences between Earth and Mars are a consequence of the material which accreted to form the two planets and which are due to the physical conditions (e.g., pressure, temperature, and oxygen fugacity) present during planetary differentiation e.g., refs. 37 and 29.

## Conclusions

We have detected core transiting phase SKS from two distant events on Mars. The travel times of these phases, relative to travel times of mantle-sensitive signals, are used to produce models of the seismic properties throughout Mars and represent the first interior models that are informed by core-transiting seismic waves. Our observations provide the first direct constraints on the elastic properties of the Martian core. We find that the median seismic velocity at the top of the Martian core is ∼4.9 to 5.0 km/s, with the precise value depending on the mantle-sensitive seismic data, assumed mantle composition, and inversion methods employed. The seismically determined density and velocity, estimated without any a priori assumption on composition, can be compared to those of a Fe–O–S–C–H alloy at Mars’ core conditions to constrain the abundance of light elements. This comparison yields further evidence in favor of a high fraction of light elements alloyed with iron, independently of the inversion method, mantle chemistry, and mantle travel-time dataset used. Future geophysical missions to Mars will be vital to refining models of the Martian core beyond these first seismological estimates, and a multilocation network of seismometers (61) may prove critical to enhancing our knowledge of Mars’s deep interior. Meanwhile, continued analysis of the InSight seismic data will prove helpful in further refining models of Mars’ interior structure.

## Materials and Methods

We used seismic data from the InSight mission in this research. InSight wave-form data and the Mars Quake Service catalogue, which contains details of all events and phase picks for events up to the end of June 2022, are available from the IRIS-DMC, NASA-PDS, SEIS-InSight data portal, and IPGP data center (47, 91, 92). SKS signals were picked using five different methods, labeled methods A–E in this paper. Full details of the processing steps needed are described in *SI Appendix*, section S2. Two different inversion schemes were used to obtain models of Martian density and seismic velocities. Detailed descriptions of these schemes, which follow the methods of refs. 7 and 8, are provided in *SI Appendix*, section S4. Details of the compositional inversions are also given in *SI Appendix*, section S4. This work used software packages GMT (93), matbplotlib (94) and ObsPy (95).

## Supplementary Material

Appendix 01 (PDF)

## Data Availability

Seismic data have been deposited in IRISDMC, NASA PDS, IPGP Data Center Services (https://doi.org/10.18715/SEIS.INSIGHT.XB_2016).
