# Supplementary material for: First observations of core-transiting seismic phases on Mars
Source: Proc Natl Acad Sci U S A. 2023 Apr 24;120(18):e2217090120. doi: 10.1073/pnas.2217090120 (PMC10161042; doi:10.1073/pnas.2217090120)
Supplement: Supplementary file 1 — Appendix 01 (PDF) [file pnas.2217090120.sapp.pdf]

SUPPLEMENTARY MATERIAL

First observations of core transiting seismic phases on Mars

Jessica C.E. Irving, Vedran Lekić, Cecilia Durán, Mélanie Drilleau, Doyeon Kim, Attilio Rivoldini, Amir Khan, Henri Samuel, Daniele Antonangeli, William Bruce Banerdt, Caroline Beghein, Ebru Bozdağ, Savas Ceylan, Constantinos Charalambous, John Clinton, Paul Davis, Raphaël Garcia, Domenico Giardini, Anna Catherine Horleston, Quancheng Huang, Kenneth J. Hurst, Taichi Kawamura, Scott D. King, Martin Knapmeyer, Jiaqi Li, Philippe Lognonné, Ross Maguire, Mark P. Panning, Ana-Catalina Plesa, Martin Schimmel, Nicholas C. Schmerr, Simon C. Stähler, Eleonore Stutzmann, Zongbo Xu

Contents

List of Figures **2**

1 Overview **2**

2 Observations **2**
2.1 Summary of observational methods . . . 2
2.2 Method A . . . 3
2.3 Method B . . . 7
2.4 Method C . . . 10
2.4.1 S1000a . . . 10
2.4.2 S0976a . . . 10
2.5 Method D . . . 13
2.6 Method E . . . 13
2.7 Environmental noise analysis . . . 13
2.8 S0976a: Note on the signal $\sim$ 250 s after PP . . . 17

3 Synthetic modelling **18**
3.1 Martian travel time curves . . . 18
3.2 Ray theoretical predictions: event location . . . 18
3.3 Ray theoretical predictions: changing only Mars' core . . . 19
3.4 Waveform modelling of relative amplitudes . . . 20

4 Inversions **23**
4.1 Geodynamical inversions . . . 23
4.1.1 Inversion parameterisation and methods . . . 23
4.1.2 Inversion results . . . 24
4.2 Geophysical inversion . . . 26
4.2.1 Model parameterization . . . 26
4.2.2 Inverse problem . . . 27
4.3 Full seismic models . . . 29
4.4 Equation of state for the liquid core . . . 29
4.5 Core composition inference . . . 30
4.5.1 Extended analysis of seismic models of inversions . . . 31

Bibliography **32**

List of Figures

S1 Schematic illustration of the seismic analysis methods 4
S2 Seismic data and frequency-dependent polarization analysis for S0976a 5
S3 Seismic data and frequency-dependent polarization analysis for S1000a 6
S4 Polarization analysis of S0976a waveforms. 8
S5 Polarization analysis of S1000a waveforms. 9
S6 PP, SKS and SS picks: Method C 11
S7 PP and SS picks: Method C 12
S8 Environmental noise around SKS: S0976a 15
S9 Environmental noise around SKS: S1000a 16
S10 Glitch at $\sim 250s$ after PP 17
S11 Travel time curves for Mars 18
S12 Impact of changing hypocentre 19
S13 Models with core velocity and gradient varied 19
S14 Impact of changing Mars' core velocity 20
S15 Expected phase amplitudes for S0976a 21
S16 Expected phase amplitudes for S1000a 22
S17 Marginal distributions using the geodynamical inversion approach 25
S18 Geodynamical inversion results using the EH45 and YMD mantles 25
S19 Marginal distributions for CMB radius using the classical parameterization 25
S20 Fit to seismic data of the geodynamical models 26
S21 Full-planet velocity and density models 29
S22 Effect of light element concentration on density and acoustic velocity 30
S23 Density-velocity relations for different light element combinations 31
S24 S and H posteriors for geodynamical model 32

1 Overview

This supplement contains additional information on the five different picking methods used (Section 2), synthetic modelling (Section 3) and seismic inversion and compositional inference methods (Section 4).

2 Observations

2.1 Summary of observational methods

On Earth, routine observations of SKS signals are not made for earthquakes with magnitudes as low as those detected on Mars. Despite the lower noise levels on Mars [1], SKS is not readily visible in the three-component seismograms. Thus we have applied multiple different processing techniques to enable measurement of the SKS differential travel times for S0976a and S1000a. The methods are schematically represented in Fig. S1 and can be grouped into two categories: arrival detection based methods and cross correlation based methods. The former category applies signal processing techniques to the waveforms (including polarization filtering and analysis in the frequency domain). Methods in the later category use mantle-transiting waves as a template to match the shape of the SKS arrival, which also serves as extra confirmation that we have identified the correct signal. Sections 2.2-2.4 detail the three arrival based detection methods while Sections 2.5-2.6 explain the cross correlation-based methods we use.

In an environment with no seismic noise, straightforward source-time functions and precisely identifiable backazimuths, one would expect methods A-E to all give the same differential travel time. However the challenging Martian data contain frequency-dependent environmental noise (see Section 2.7) and for S0976a the precise location of the event has uncertainty associated with it [2]. The second rows of Figures S2 and S3 show the bandpass filtered data for the two events rotated into vertical, radial and transverse components. The signals are noisy, especially in the SKS and SS windows which contain considerable energy throughout. Thus the five different methods result in measurements which have standard deviations of 4-8 s (main paper

Table 1). This range helps us to understand the uncertainty associated with estimating the differential travel times. The spread of measurements will be impacted by the differences between the techniques used. In particular, different filters and/or windowing choices are compounded by other features – for example the specific way that the polarization analysis is conducted will further enhance features at different frequencies, and so they have used different optimal frequency ranges.

Three of the methods used (A–C) seek to find the arrivals of the PP, SKS and SS waveforms at InSight. Though all three employ polarization as part of their workflows, they make different processing choices to obtain their final results. Polarization filtering itself is a non-linear process and details of the calculations – for example using the S-transform or short-time Fourier Transform – impacts the frequency window at which SKS (as well as PP and SS) is clearest and therefore the precise details of the picks. Two of the methods (D & E) instead rely on cross-correlation of the seismic record. Additionally, the presence of well-documented precursors to the mantle transiting phases [3] can affect the precise differential travel time measurements.

Cross-correlation Method D uses an amplitude-insensitive measure and is therefore naturally sensitive to different frequencies of energy to the amplitude-sensitive arrival detection methods A-C. The two different cross correlation methods use different components for their SS templates: Method D uses the radial component, ensuring that the source-side interactions are the same, while Method E uses the transverse component as the SS onset is clearer to identify. These different, but we believe equally reasonable, choices will also lead to different parts of the SKS signal being highlighted. Our environmental noise analysis (Figs. S8 and S9) is performed across a range of frequencies to show variations in the relative amplitudes of potential wind contamination. The SKS signal is above the noise level at relevant frequencies in this analysis.

We do not prefer any one method over the others - they each make choices which have emphasized different features of the data (both in the SKS signals and in the PP and SS signals used for reference). We note that the two cross-correlation methods do not produce results which are systematically different to the arrival detection methods, even though the cross correlation methods focus on matching phase. We provide for completeness an explanation of why we do not pick the signal which arrives ~ 250 s after the PP signal – it is a mild glitch – in Section 2.8.

2.2 Method A

We use the seismic recording of S0976a and S1000a, the two distant marsquakes ($> 100°$) detected by InSight [2]. The 20 samples per second UVW channel data from the Very Broad Band (VBB) sensor of the Seismic Experiment for Interior Structure (SEIS) is rotated to ZNE. Glitches are removed based on [4] to prevent potential pitfalls in the analysis and interpretation of the SEIS data [5]. We conduct frequency-dependent polarization analysis (FDPA) on the S0976a and S1000a waveforms to identify the arrival of core-traversing phase SKS (Fig.1).

We start by computing the S-transform of three-component event waveforms and compute a 3x3 cross-spectral covariance matrix in 90% overlapping time windows whose duration varies inversely with frequency [e.g. 6]. The relative sizes of the eigenvalues of this covariance matrix are related to the degree of polarization of the particle motion, while the complex-valued components of the eigenvectors describe the particle motion ellipsoid in each time-frequency window. We search for seismic arrivals of rectilinear particle motion dominantly polarized in vertical (VRM) and horizontal directions (HRM) by combining polarization attributes resulting from the FDPA [e.g., see supplementary material in 7]. This approach has been previously implemented to highlight and identify other body and surface wave phases such as ScS [7] as well as minor-arc Rayleigh waves on Mars [8]. Two distinctive arrivals for S0976a and S1000a (Figs. S2 and S3 and main paper Figure 1C) are identifiable based on the largest difference between the average HRM and VRM values, summed across the 0.3 – 0.8 Hz frequency band, within the SKS prediction windows. These prediction windows are created by allowing core velocity to vary by ±10% from the InSight_KKS21_GP model.

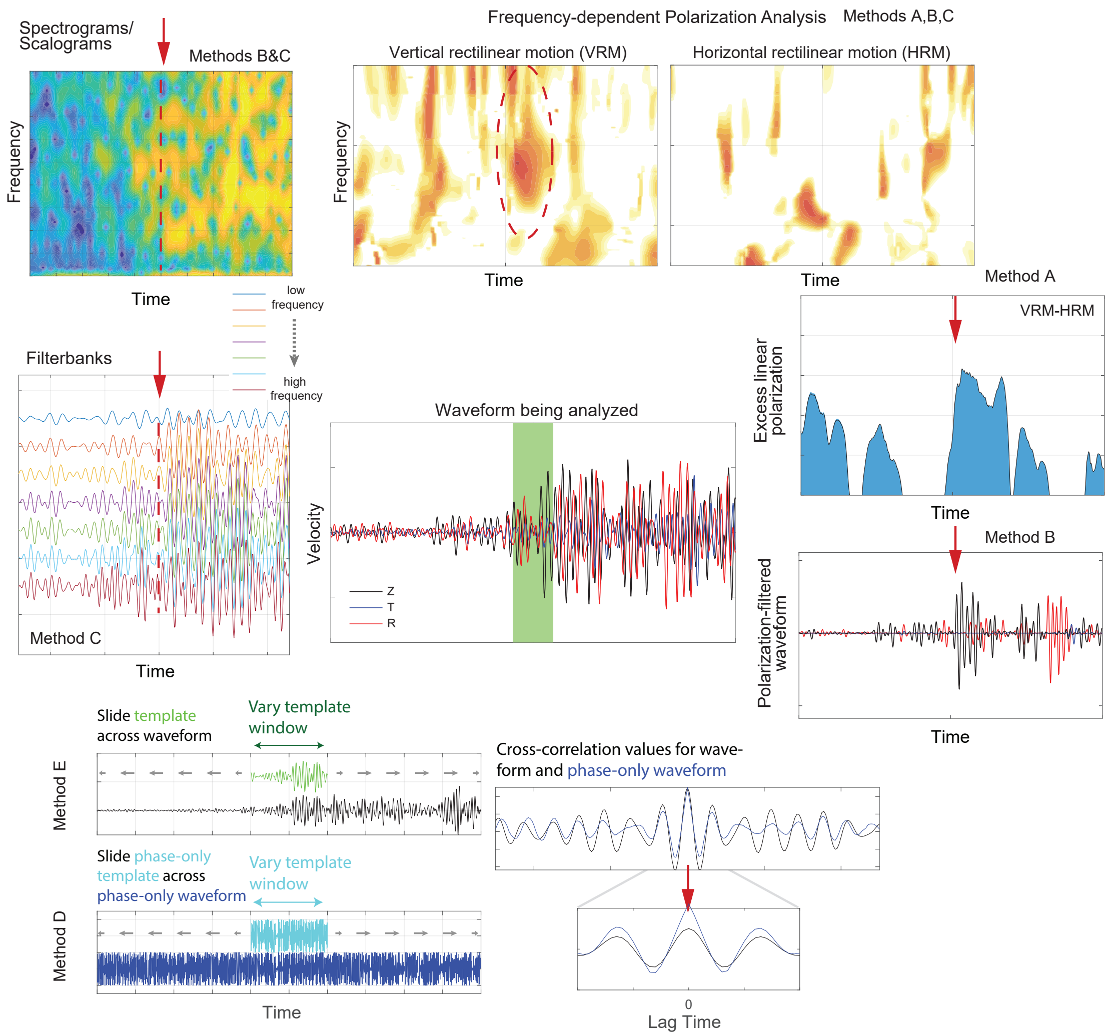

Figure S1: Schematic illustration of the seismic analysis methods we employ utilize the three-component waveforms to identify arrivals either by their amplitude content or polarization attributes, or by using template cross-correlation. Methods A, B, and C seek arrivals with strong rectilinear polarization, either by looking at the excess of horizontally- or vertically-polarized rectilinear motion (method A) or polarization-filtered waveforms (method B). Methods B and C combine polarization analyses with spectrogram/scalogram analysis and/or filterbanks. Finally, methods D and E primarily rely on phase cross-correlations (method D) and standard cross-correlations (method E) between template waveforms and the target waveforms.

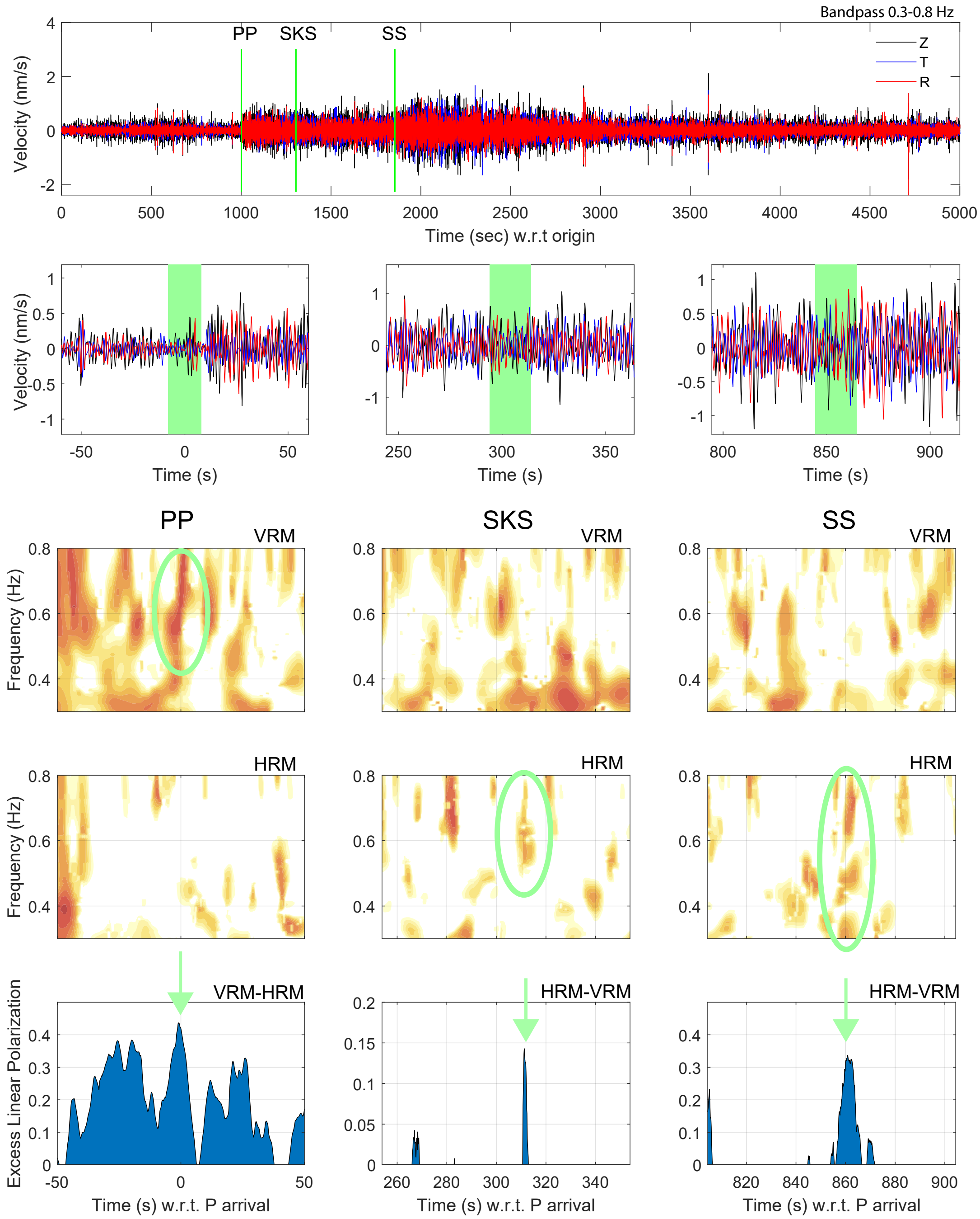

Figure S2: Seismic data and frequency-dependent polarization analysis for the S0976a (Method A). Top row: Vertical (Z), radial (R) and transverse (T) bandpass filtered seismograms (0.3-0.8 Hz) for event S0976a. Second row: Seismograms from the top row windowed around the arrivals of PP (left), SKS (center) and SS (right). Third row: Vertical component of FDPA analysis for PP, SKS and SS. PP is a vertically polarized signal (circled). Third row: Horizontal component of FDPA analysis for PP, SKS and SS. SS and SKS are a horizontally polarized signals (circled). Bottom row: Excess linearly polarized energy for PP, SKS and SS.

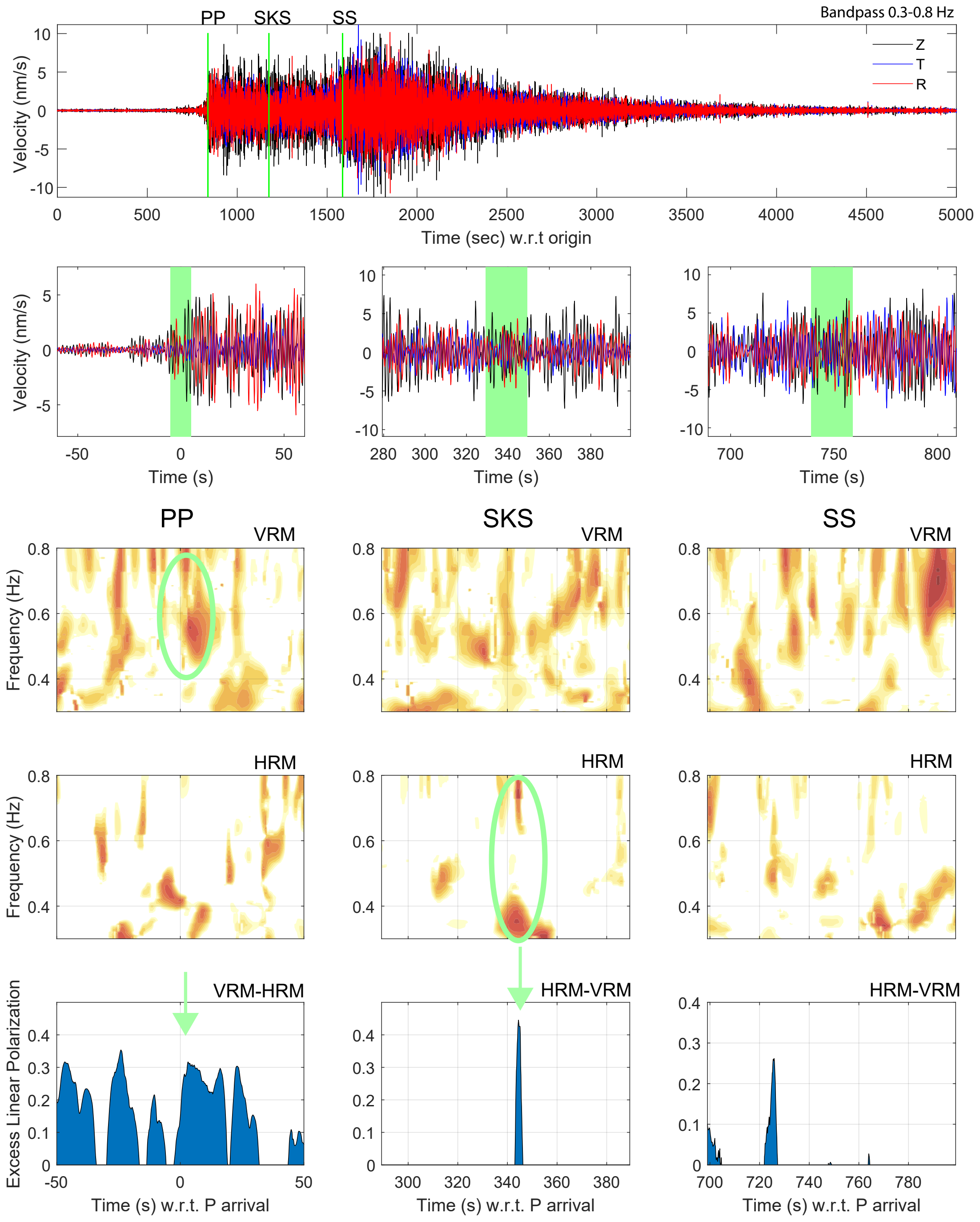

Figure S3: Seismic data and frequency-dependent polarization analysis for S1000a (Method A). Top row: Vertical (Z), radial (R) and transverse (T) bandpass filtered seismograms (0.3-0.8 Hz) for event S1000a. Second row: Seismograms from the top row windowed around the arrivals of PP (left), SKS (center) and SS (right). Third row: Vertical component of FDPA analysis for PP, SKS and SS. PP is a vertically polarized signal (circled). Third row: Horizontal component of FDPA analysis for PP, SKS and SS. SS and SKS are a horizontally polarized signals (circled). Bottom row: Excess linearly polarized energy for PP, SKS and SS.

2.3 Method B

We employed a polarization filter method [9] to pick the PP, SS and SKS phases in the S0976a and S1000a events. This method has been successfully used to detect body waves such as surface-reflected waves [10], mantle triplications [11] as well as core-reflected waves [7] from InSight seismic data. The details of this method can be found in the supplements of [10] and [7]. We first removed the instrument response from the deglitched data [4] and rotated the UVW components to the ZNE components. We applied a Butterworth bandpass filter to filter the S0976a and S1000a events between 0.15-0.8 and 0.3-0.9 Hz, respectively. We then applied the polarization filter in the ZNE coordinate, and picked PP arrivals on the peaks of the polarization filtered vertical envelopes (Fig. S4a and Fig. S5a). For S0976a event, we constrained the back-azimuth from the polarization of PP phase Table S1. S1000a event is confirmed to be a meteoroite impact [12]. Therefore, we used the impact location (38.425°N, 79.969°W) to calculate the back-azimuth and epicentral distance of this event (Table S1). We rotated the horizontal components to the radial (BHR) and transverse (BHT) components using the pre-calculated back-azimuths.

Event	SKS-PP Time (s)	SS-PP Time (s)	SS-SKS Time(s)	Distance (°)	Backazimuth (°)
S0976a	298.3 ± 25.0	853.4 ± 15.0	555.1 ± 20.0	145.0 ± 3.5	101.8 ± 3.8
S1000a	334.8 ± 18.0	752.3 ± 16.0	417.5 ± 18.0	125.9	34.2

Table S1: Summary of body wave picks, epicentral distances and backazimuths of S0976a and S1000a events from Method B. The epicentral distance and backazimuth of S1000a event are determined from the impact location [12] so no uncertainties are assigned.

We applied the polarization filter again in the ZRT coordinate to pick the SS and SKS phases. We picked the SS phase of S0976a on the BHT component to avoid the S-to-P conversions Fig. (S4c). We then constrained the epicentral distance of S0976a event (Table S1) from the SS-PP differential time based on the InSight_KKS21_GP model [10, 13, 7]. Given the epicentral distance, we calculated the predicted travel time of SKS phase using the InSight_KKS21_GP model and assuming a source depth of 30 km. Due to the uncertainties of the P-wave velocities in the model, we picked the SKS arrival on the BHR component within 20s of the predicted travel time (S4b). Note that a small glitch occurred before the SKS arrival and was dominated by low-frequency energy. Therefore, we bandpass filtered the SKS phase between 0.3-0.9 Hz to suppress the glitch in this time window. Unlike S0976a, we were able to use the known epicentral distance (125.9°) and source depth (0 km) of S1000a to calculate the predicted travel times of SKS and SS phases for this event. We picked the SKS arrival on the BHR component within 20 s of the predicted travel time (Fig. S5b). Since S1000a is an impact event, the SS phase is predominant on the BHR component. We filtered the SS phase between 0.3-0.8 Hz to suppress high-frequency noise and picked this arrival on the BHR component within 10 s of the predicted travel time (Fig. S5c).

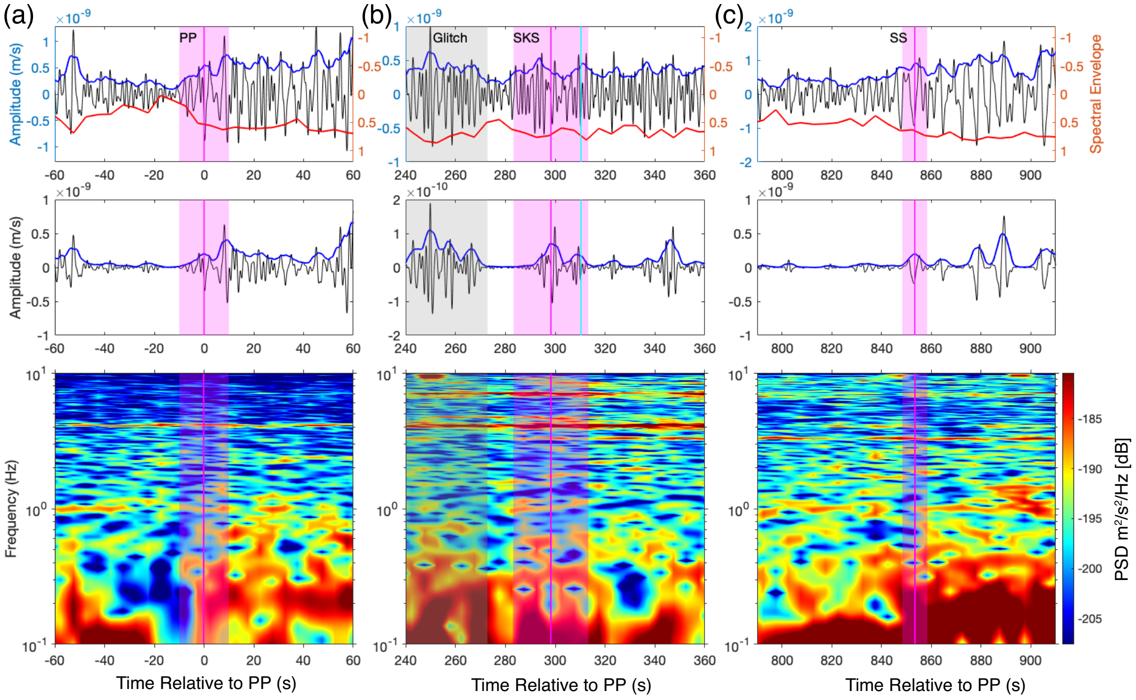

Figure S4: Bandpass filtered waveforms (top), polarization filtered waveforms (middle) and spectrograms (bottom) for S0976a (Method B). (a) PP phase on the vertical component, (b) SKS phase on the radial component, and (c) SS on the transverse component. The first row shows the bandpass filtered waveforms in black and the corresponding envelopes in blue. PP and SS phases are filtered between 0.15-0.8 Hz. SKS is filtered between 0.3-0.9 Hz to suppress the earlier glitch. The spectral envelopes, which are computed from the average amplitudes on the spectrograms within the frequency band of the corresponding bandpass filters, are shown in red with a reversed vertical axis. Pink lines and pink shaded regions represent the body wave picks and their uncertainties, respectively. The uncertainties for PP, SKS and SS picks are 10 s, 15 s and 5 s, respectively. The cyan line indicates the predicted SKS travel time using the InSight_KKS21_GP model [10, 13, 7] for a 30 km depth source with a 145-degree distance. The gray shaded region indicates the instrument glitch. The second row shows the polarization filtered waveforms in black and the corresponding envelopes in blue.

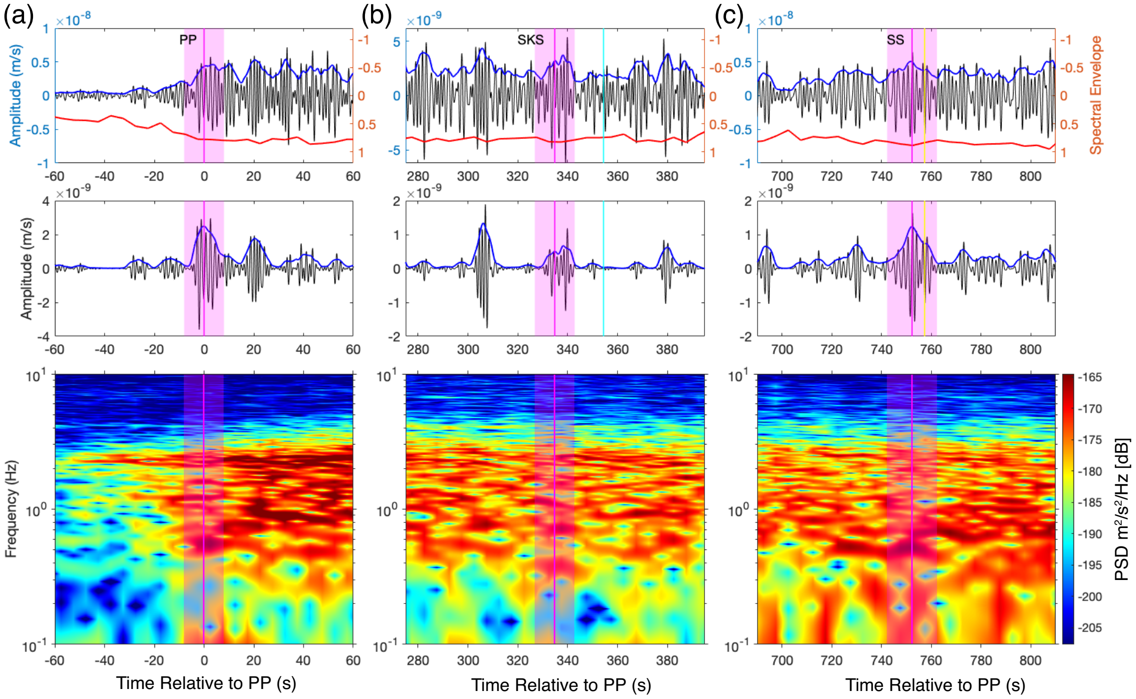

Figure S5: Bandpass filtered waveforms (top), polarization filtered waveforms (middle) and spectrograms (bottom) for S1000a (Method B). (a) PP phase on the vertical component, (b) SKS phase on the radial component, and (c) SS on the radial component. The first row shows the bandpass filtered waveforms in black and the corresponding envelopes in blue. PP and SKS phases are filtered between 0.3-0.9 Hz. SS phase is filtered between 0.3-0.8 Hz. The spectral envelopes, which are computed from the average amplitudes on the spectrograms within the frequency band of the corresponding bandpass filters, are shown in red with a reversed vertical axis. Pink lines and pink shaded regions represent the body wave picks and their uncertainties, respectively. The uncertainties for PP, SKS and SS picks are 8 s, 8 s and 10 s, respectively. The cyan and yellow lines indicate the predicted SKS and SS travel times, respectively, using the InSight_KKS21_GP model [10, 13, 7] for a 0 km depth source with a 125.9-degree distance. The second row shows the polarization filtered waveforms in black and the corresponding envelopes in blue.

2.4 Method C

Following the procedure described in [14], we apply complementary approaches to identify body-wave phases in events S0976a and S1000a: time-domain envelopes, filter banks, and polarized waveforms. Building upon the initial identification in the MQS Catalog [15], we identify and refine the PP and SS body-wave picks and search for core-traversing phases. The detailed analysis applied to each event is described in this subsection.

2.4.1 S1000a

Since a comprehensive description of the identification of PP- and SS-wave arrivals is given in [16], here we focus mainly on the identification of the SKS-wave arrival. Through the application of filter banks, polarized waveforms, and polarization analysis, we refined the initial picks and considerably reduced the uncertainties of [15], allowing us to properly align the event. Envelopes of the event with the PP- and SS-wave picks and their uncertainties represented as vertical lines and bars, respectively, are shown in Figure S6.

Since the location of the event is well known [2], waveforms are rotated to and envelopes are shown in the Vertical-Radial-Transverse (ZRT) system. Grey curves in Figures S6A–C represent the time-domain envelopes of band-pass filtered waveforms (0.2–0.5 Hz), while blue curves represent envelopes of the waveforms after applying a time-domain polarization filter [9]. Because body waves exhibit a high degree of linear polarization, the application of the polarization filter increases the signal-to-noise ratio of the linearly polarized part of the signal.

Figure S6D shows a three-component scalogram of the event computed as the absolute value of the continuous wavelet transform of the signal. This scalogram, built as the sum of the squared scalograms of each component, illustrates the change in frequency content of the signal with time. While the main PP- and SS-wave arrivals are discernible in the envelopes and scalogram, on account of the broad frequency content of S1000a, and the interference of numerous phases after the PP-wave arrival, the identification of additional phases in the PP- and SS-wave coda is quite challenging, as observed in Figure S6. In addition, non-seismic signals such as the large glitches visible in Figure S6D, including smaller glitches (indicated by the orange box), contaminate the signal, making the correct identification of seismic phases difficult. Consequently, predictions of the arrival times of body-wave phases are essential for their positive identification [see 14, for details].

To narrow the search for the SKS phase, we predict the arrival time of SKS by considering the Martian seismic models of [14] and perturbing the P-wave velocity in the core by $\pm15\%$ to account for the lack of current information on seismic velocities in the core. For S1000a, the so-predicted time range is represented by the green shaded area in Figures S6A–C. Due to the nature of SKS, we expect to see it in the radial (R) component. While the time-domain envelopes of the non-polarized waveforms do not exhibit any particularly distinctive arrival within the expected time range, a clear peak is discernible around 340 s in the R component of the polarized waveform envelopes. This large-amplitude arrival, observed to be strongly linearly polarized, is consistent with synthetically-computed SKS waveform amplitudes. No other large-amplitude arrival compatible with the SKS characteristics is discernible within the expected time window.

As a means of validating the selected seismic phase, we assessed the velocity traces in narrow frequency bands, allowing us to better understand the partitioning of energy across different frequencies. We use a bandwidth half an octave wide around each central frequency, ranging from 1/5.7 to 1/1.4 Hz. The selected filter banks cover the frequency bands where the low-frequency energy of the event is most visible and is mainly devoid of glitch-, donk- or atmospheric-related artefacts [4, 5]. This analysis demonstrated the presence of a strongly polarized arrival at frequencies ranging from 1/2.8 to 1/1.4 Hz that conforms with our established criteria for identifying seismic phases [14] and that we identify as the SKS phase.

2.4.2 S0976a

Analogous to the analysis presented for event S1000a, Figure S7 shows envelopes of polarized (blue) and non-polarized (grey) waveforms in the vertical-radial-transverse (ZRT) system (Figure S7A–C) and three-component scalogram (Figure S7D) of event S0976a. While an estimate of the back azimuth for event S0976a based on the observed particle motion of the PP-wave arrival is available [2], the uncertainty is considerable ($\pm25^{\circ}$), and rotation of the waveforms does not demonstrably improve detection of SKS.

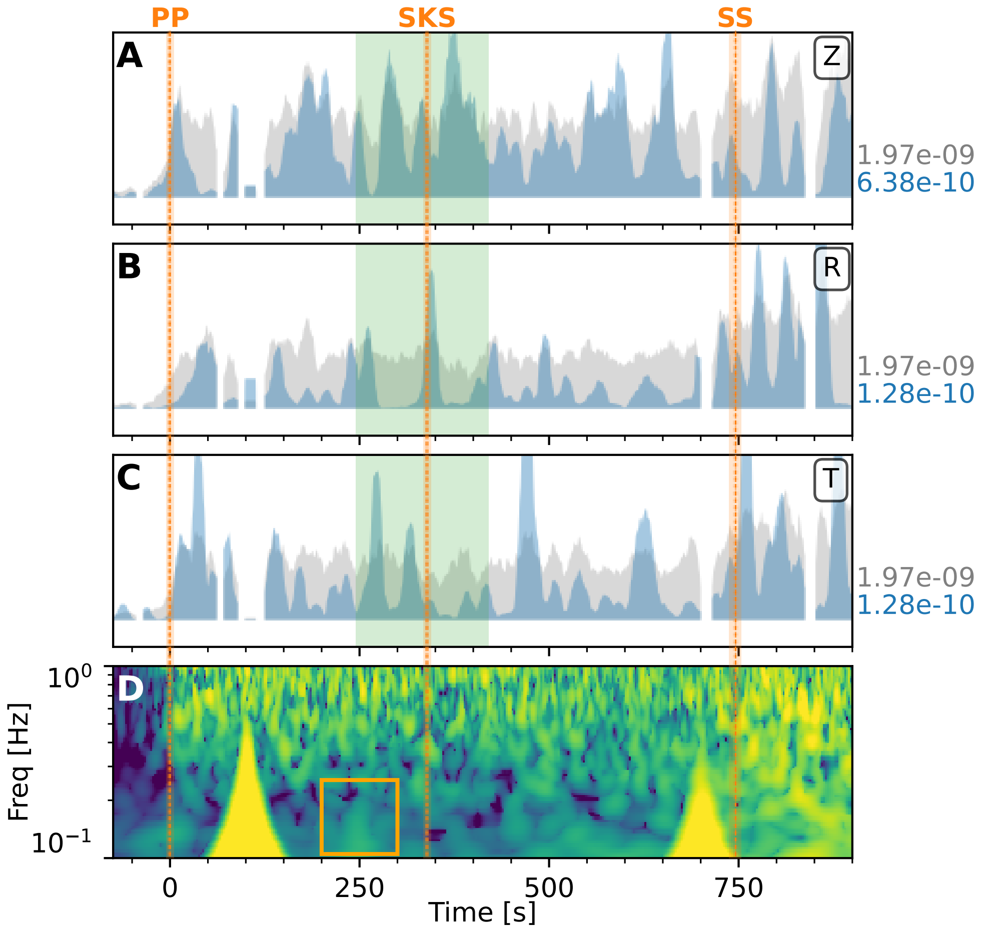

Figure S6: Time domain envelopes of band-pass filtered (0.2–0.5 Hz) polarized (blue) and non-polarized (grey) waveforms of (A–C) and three-component scalogram (D) of event S1000a (Method C). Phase picks for S1000a and their uncertainties [16] are shown as vertical solid lines and orange bars, respectively. The normalisation factors applied to each envelope are shown in their corresponding colour to the right of each panel. Envelopes are masked where glitches occurred to avoid misinterpretation of phases. The green band represents range for expected arrival of SKS based on inverted models where the core velocities were perturbed ±15%. The orange box in (D) indicates the presence of a small glitch (see Section 2.8), the large yellow wedges correspond to two large glitches. Envelopes are normalized for visual purposes. Time is relative to the arrival of the PP wave.

In contrast to S1000a, S0976a has a much more impulsive PP-wave arrival, allowing us to easily pick it in the band-pass filtered (0.1–0.5 Hz) polarized and non-polarized waveforms. In addition, its arrival is clearly discernible in the scalogram, characterized by a considerable increase of energy. For the SS-wave arrival, due to the presence of a small glitch which affects the waveforms, we mainly rely on the scalogram to first identify the energy onset. Both, PP- and SS-wave picks are represented as vertical lines in Figure S7. While the increase of energy is clear in the scalogram for both PP- and SS-waves, the picks also coincide with the break of the slope in the Z-component envelopes for PP and the horizontal components for SS. However, due to the presence of several glitches close to the PP- and SS-wave arrivals and the windy conditions that are present throughout the event [2], we assign larger uncertainties to both picks (represented by the vertical bars in Figure S7) in comparison to event S1000a.

Because S0976a has not been reliably located, the prediction of a potential SKS phase relies on the alignment of the event through PP- and SS-wave arrival times. We follow the procedure described in the

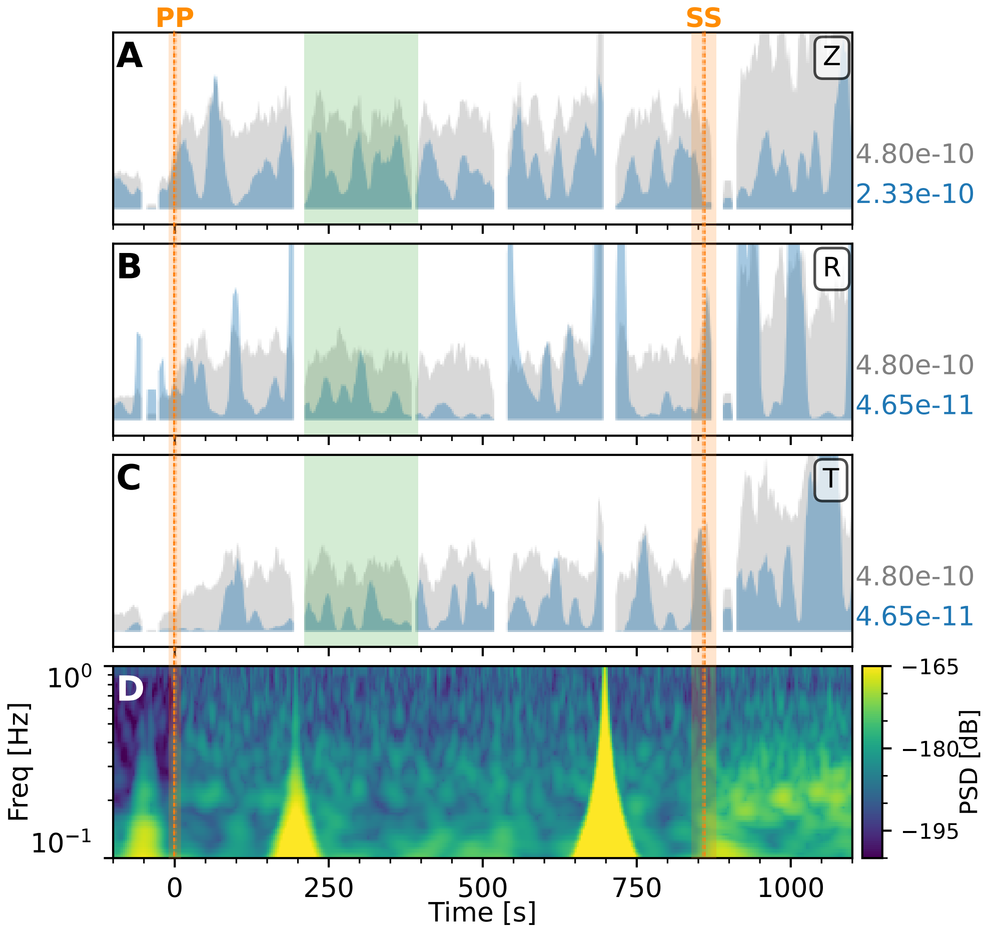

Figure S7: Time domain envelopes of band-pass filtered (0.2–0.5 Hz) polarized (blue) and non-polarized (grey) waveforms of (A–C) and three-component scalogram (D) of event S0976a (Method C). Phase picks for S0976a and their uncertainties [14] are shown as vertical solid lines and orange bars, respectively. The normalisation factors applied to each envelope are shown in their corresponding colour to the right of each panel. Envelopes are masked where glitches occurred to avoid misinterpretation of phases. The green band represents range for expected arrival of SKS based on inverted models where the core velocities were perturbed ±15%. The three large yellow wedges correspond to large glitches.

previous section and compute a range of times that allow us to narrow the possible arrival time of SKS. In comparison to S1000a, however, a single strong SKS candidate is not apparent, but a series of similar-amplitude and strongly linearly-polarized arrivals in the predicted time window for SKS are present. The lack of a clear candidate for SKS might be explained by 1) lack of an accurate location that would enable perfect rotation of the traces into the ZRT system and 2) the noisy (wind) conditions that characterises the event. The lack of a precise location leads us to analyse both North and East components. The time window during which SKS is expected contains four linearly-polarized peaks. Of the possibilities, we can rule out the earliest because it would correspond to an SKS arrival that is too early relative to what we would expect based on S1000a. Of the remaining arrivals we favour the signal at ∼310 s since it appears on both N and E components and is present when narrow-band filtered at both 1/2 and 1/1.4 Hz; yet we have to acknowledge the uncertain nature of the pick. Our final PP, SS, and SKS picks, selected at the onset of energy of the corresponding phases are listed in Table S2.

Table S2: Absolute observed PP-wave arrival time picks and body-wave differential travel times for SS-PP, and SKS-PP from Method C.

Event	T_{PP}	T_{SS}-T_{PP}	T_{SKS}-T_{PP}
S0976a	2021-08-25T03:49:01 (±8 s) UTC	859.0 s (±20 s)	*310 s (±8 s)**[*]
S1000a	2021-09-18T18:01:57 (±5 s) UTC	745.6 s (±8 s)	339 s (±5 s)

[*]selected pick is uncertain

2.5 Method D

We search for SKS signals using a multi-template correlation approach as already employed to find core reflections in the S-wave coda of Martian events (ScS signals, Figs. S3-1 and S3-2 in [7]) and Earth upper mantle reverberations/conversions [17]. For SKS detection, our templates include the SS arrival as the direct S phase is in the core shadow owing to the large epicentral distance for these events. Here, we employ 13 templates for seven different one-octave frequency bands. Templates start 5 s before SS and have variable length ranging from 20 to 190 s (20, 25, 30, ..., 100, 130 160 190 s). Short templates include only the SS arrival while longer templates permit to include also source-site reverberations which can increase the SNR of the correlation. A welcomed side-effect of long templates is the attenuation of correlation cross-terms [18]. Further, after extensive testing, we decided to work with seven 50-percent overlapping band-pass filters with one-octave band-width between 0.05 and 0.8 Hz. The main purpose of using different frequency bands is to identify SKS through its stability over a broad frequency band. Finally, we perform a Hilbert transform of the template to account for the 90 degree phase shift of the SS-waveforms with respect to SKS. The waveform distortion is caused by an internal caustic of the SS ray path. The templates are correlated with the PP coda time window on the three components using the phase cross-correlation [PCC, 17]. PCC is based on analytic signals and provides signal amplitude unbiased results as shown in many applications [e.g. 19, 20, 21]. The SKS identification is done on the radial component.

The horizontal components of the S0976a event are not rotated as the East component is within the error bounds of the identified back azimuth of 101°. For S1000a, the horizontal components are rotated by 52° to obtain radial and transverse components. The multi-template correlation approach has not been conclusive for the S1000a event while for the S0976a event three possible SKS phases have been identified by their robust correlation. The signals manifest strongest between 0.1 and 0.4 Hz and their travel times with respect to the SS phase are −546 s, −529 s and −505 s. Error bounds are safely within ±5 s and the largest error source is the mis-identification of SKS.

2.6 Method E

We pick the SKS signal from S0976a using waveform matching [10] and polarization analysis [e.g. 22, 7]. We bandpass filter the Z/N/E-component seismic recordings between 0.2 and 0.6 Hz; we then rotate the N and E components to the radial and transverse components. Waveform matching requires a waveform template and we choose the template from the transverse component to avoid the potential S-to-P conversion priori to the SS arrival at the radial component [13]. The template is the Hilbert transform of a 22.5-s-long time window starting at the SS arrival time. We then cross-correlate the template with the radial-component data from 200 to 400 s after the PP arrival time to compute the cross-correlation coefficient. We also change the template length to 10 s and 16.25 s and repeat the above process. The peaks of the three sets of the cross-correlation coefficients are coincident at several time points, which means that the waveforms at these time points are robustly similar to the SS waveform. We then check the polarization at these time points and pick the arrival time, 297.4 s after the PP, where the incident-angle estimate is close to 90° from the vertical direction and the azimuth estimate is close to the S0976a backazimuth, 101° from North.

2.7 Environmental noise analysis

While InSight's seismometers are deployed on the surface underneath a Wind and Thermal Shield (WTS), a large part of the signal they measure is due to ground motion from local atmospheric disturbances. The atmospheric energy from these disturbances injects into the signal as broadband noise, requiring in-depth

analysis to better understand the environmental contribution into the seismic record and discriminate between weather-induced and seismic signals [23]. The comodulation approach has been demonstrated to be particularly successful in identifying seismic energy that is in excess of the expected broadband noise injected from the local weather [23, 24, 25, 26]. To quantify this environmental noise injection, both pressure and wind-speed measurements by the on-deck mounted Auxiliary Payload Sensor Suite [APSS 27, 28] can be used to observe instances when the seismic signal power is above that expected by the environmental noise. In the absence of wind-speed or pressure data due to lander power constraints, the excitation of highly weather-sensitive lander modes has proven an effective atmospheric proxy in estimating this injection on Mars [23, 29].

Comodulation analysis was applied to the seismic signals of S0976a and S1000a to predict the power injected by atmospheric disturbances during the entire uncertainty window of the SKS phase picks, as indicated in Table 1.

Figure S8 shows the comodulation analysis of the SKS phase and its uncertainty window on S0976a. Clear excess seismic power, here taken as the signal variance in the radial component, is observed during the SKS window of S0976a between passbands centered at periods of 2 s to 4 s against the expected atmosphere-driven noise as measured both by the pressure sensor and atmospheric proxy estimated by the lander resonance in all three directions (ZNE). The clear divergence of the seismic signal with respect to the atmospheric power strongly supports the presence of a seismic arrival of non-atmospheric origin during the SKS window. For periods ≤ 1 s and ≥ 8 s, the broadband noise injected by the atmosphere dominates and the strength of seismic signal becomes much weaker than pressure and lander-mode estimates, following closely the noise trajectory injected into the system over time.

Figure S9 shows a similar comodulation analysis of the SKS phase and its uncertainty window on S1000a. The SKS phase shows clear excess seismic power over the expected noise injected by the atmosphere. Because both wind and pressure sensors were switched off during S1000a due to power restrictions, the atmospheric injection is here estimated by the weather-sensitive lander resonance at $\sim$6.5 Hz which is outside the bandwidth of S1000a and therefore unaffected by signal emanating from the event itself. The excess seismic signal power can be observed from the passband centered at 1/4 Hz up to at least 2 Hz, strongly suggesting the presence of seismic power of non-atmospheric origin. While there is still partial excess energy at 1/5.7 Hz, the signal-power divergence begins to weaken with the passband at 1/8 Hz appearing to be strongly driven by the environment as shown by the match between the traces.

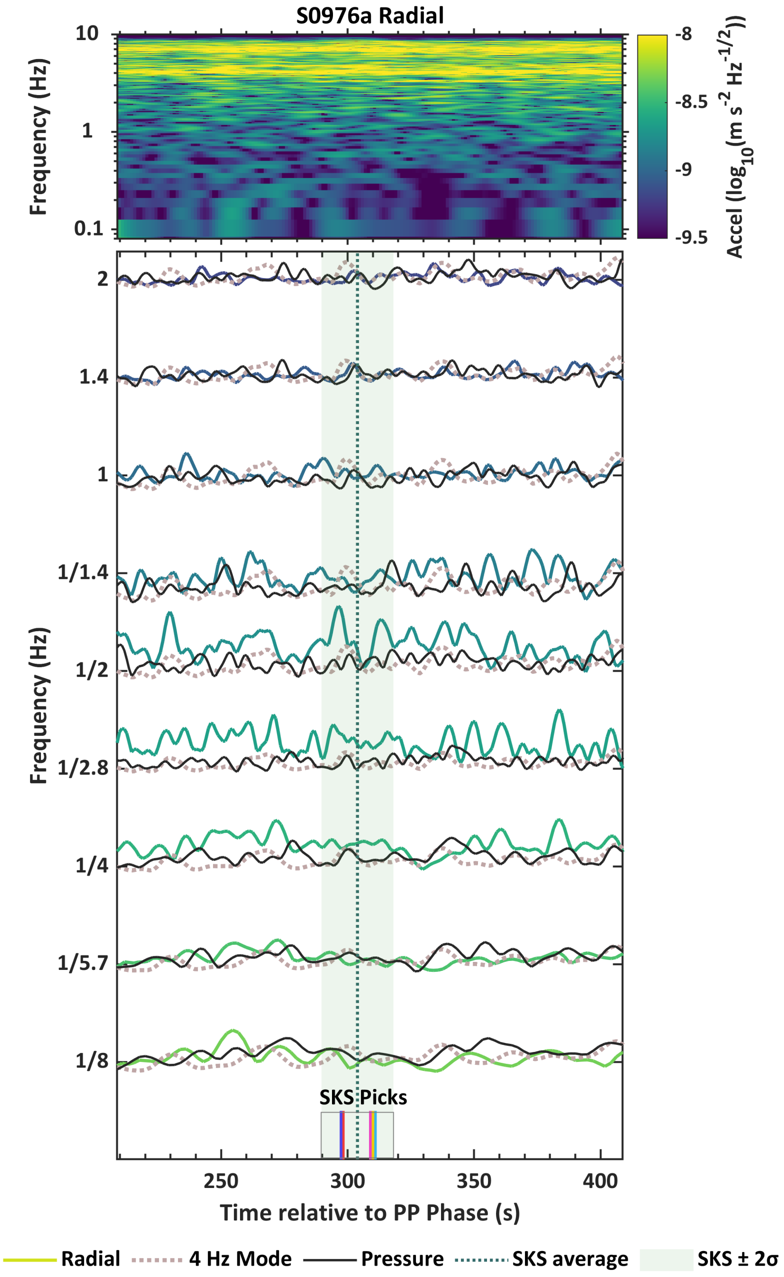

Figure S8: Comodulation analysis centered around a ± 100 s window of the average SKS pick and uncertainty window of S0976a. The top figure shows the spectrogram of the signal power in the radial VBB component at 20 sps, calculated using 25-s long Hanning windows. The line traces show a comparison between the seismic, pressure and estimated atmospheric power of the signal based on the ∼4 Hz lander resonance. The radial seismic signal power (colored lines) and atmospheric pressure power (solid black) are calculated in half-octave windows centered from 1/8 to 2 Hz. The atmospheric signal power proxy of the weather-sensitive lander resonance is calculated in the frequency range 3.7 - 4.3 Hz (dotted grey). The power of the seismic and lander resonance signal is variance- and mean-matched to the pressure signal in the window prior to the PP arrival. Where the seismic line trace is above the atmospheric and lander-resonance line traces, the residual can be attributed to seismic injection. The center of the SKS window represents the average of all reliable SKS pick observations given in Table 1 of the main text and it is marked by the vertical grey dashed line, with each individual SKS pick annotated by the coloured short vertical lines at the bottom of the figure. The shaded green region indicates a 2-σ uncertainty.

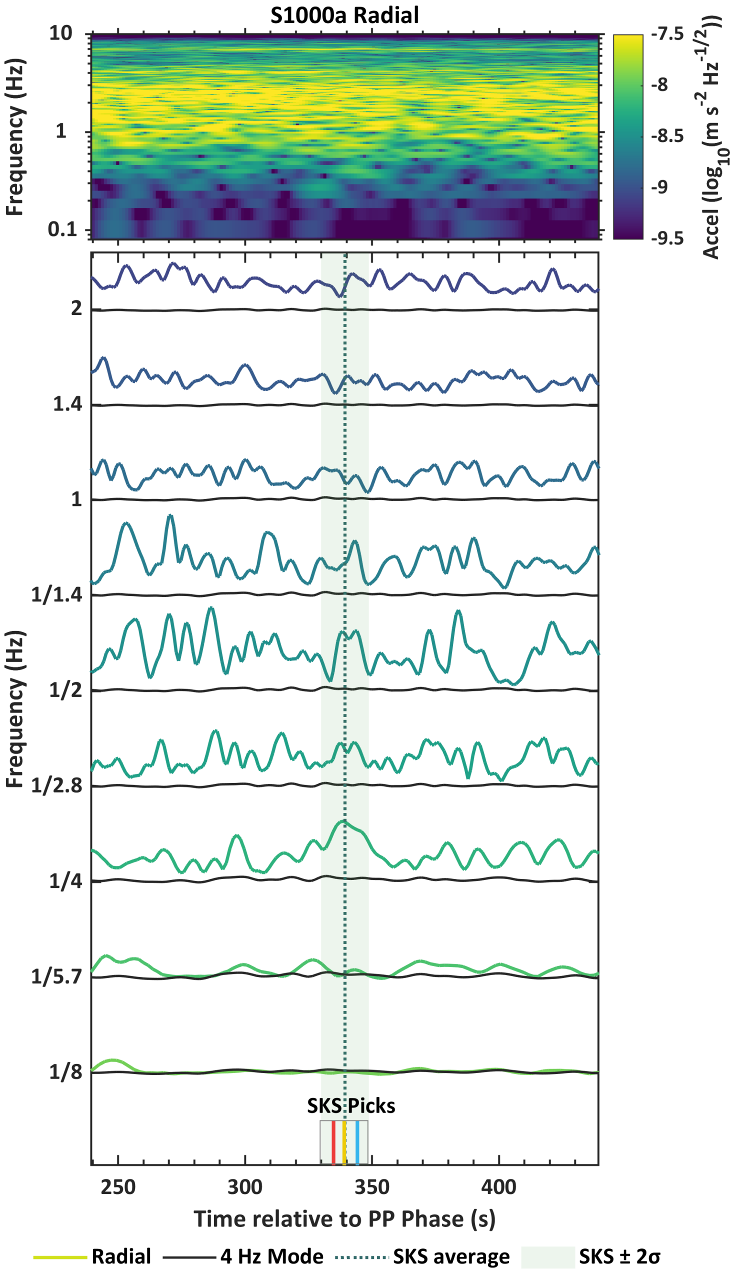

Figure S9: Comodulation analysis centered around a ± 100 s window of the average SKS pick and uncertainty window of S1000a. The top figure shows the spectrogram of the signal power in the radial VBB component at 20 sps, calculated using 25-s long Hanning windows. The line traces show a comparison between the seismic and estimated atmospheric power of the signal based on the ∼6.5 Hz lander resonance. The radial seismic signal power (colored lines) is calculated in half- octave windows centered from 1/8 Hz to 2 Hz. The proxy of the atmospheric signal power from the weather-sensitive lander resonance is calculated in the frequency range 6.5 – 7.2 Hz (solid black). The power of the seismic signal is variance- and mean-matched to the lander resonance signal in the window prior to the PP-wave arrival. Where the seismic line trace is above the lander-resonance line trace, the residual can be attributed to a seismic injection. The center of the SKS window represents the average of all reliable SKS pick observations given in Table 1 of the main text and it is marked by the vertical grey dashed line, with each individual SKS pick annotated by the coloured short vertical lines at the bottom of the figure. The shaded green region indicates a 2-σ uncertainty.

2.8 S0976a: Note on the signal $\sim$ 250 s after PP

Glitches, donks, spikes are waveform artifacts that can affect the interpretation of data from InSight's VBB seismometer [5].

Before the arrival time of the preferred SKS phase, we observe two other relatively clear signals on the radial component (i.e., indicated by the shaded regions in Fig. S10). While it could be tempting to pick one of them as the SKS phase, we ruled these signals out due to the presence of a spike and two glitches near their arrival times. Specifically, there is a spike at $\sim$ 249 s on the W component, which precedes a glitch (hereafter referred to as Glitch 1). On the U component, another glitch (hereafter referred to as Glitch 2) is visible at $\sim$ 254 s. Those two glitches are characterized by a fast rise time (onset) and a recovery (decay of their amplitudes) over 15 s. We therefore conclude that these signals (in the shaded region of Fig. S10) are likely affected by the glitches, rather than being caused by structure.

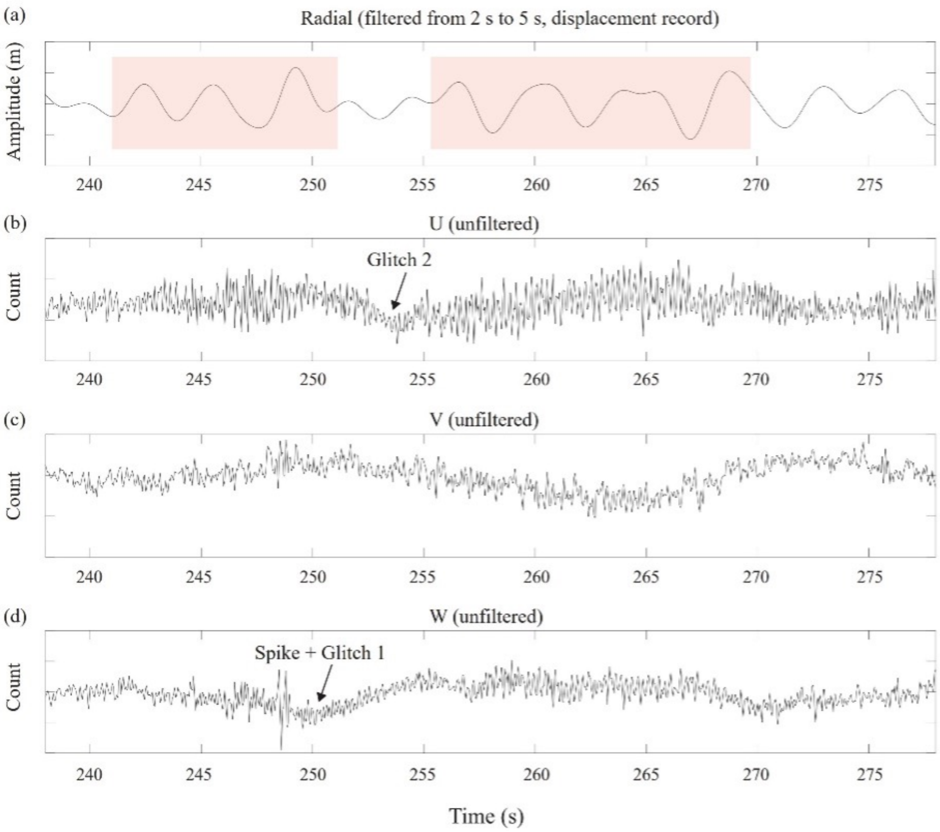

Figure S10: (a) Displacement waveform on the radial component (filtered into 2 s to 5 s). The time axis is defined after the MQS reference time (2021-08-25 03:49:06). The shaded regions indicate two suspicious signals. (b-d) The raw waveforms on the U, V, and W components, respectively. One spike and 'Glitch 1' on the W component and 'Glitch 2' on the U component are annotated.

We note that those two glitches were not listed in [2] because of the threshold choice of 0.9 used by MQS to detect glitches. Here, we were able to detect these glitches with a synthetic glitch template [4] and using a threshold of 0.84. We argue that a cross-correlation coefficient of 0.84 likely indicates a glitch since the noise level might affect the value of the cross-correlation coefficient. The cross-correlation coefficients between the data and the synthetic template were found to be 0.84 and 0.85 for Glitch 1 and Glitch 2, respectively.

3 Synthetic modelling

3.1 Martian travel time curves

Ray theoretical calculations show that the epicentral distance ranges at which difference phases can be observed on Mars are different to those of Earth. Travel time curves for mantle phases PP and SS, core-diffracted phases Pdiff and Sdiff, and core-transiting phases SKS and PKP are shown in Fig. S11.

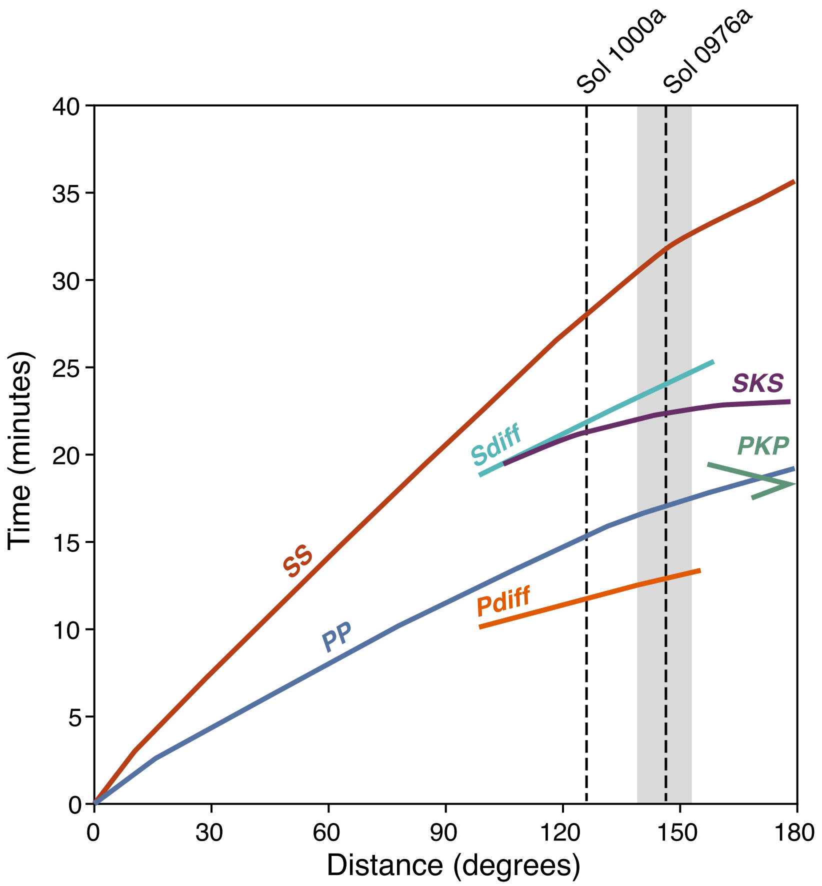

Figure S11: Travel times for selected mantle and core sensitive phases on Mars, calculated for a marsquake at the planet's surface.

3.2 Ray theoretical predictions: event location

This work employs data from two new events, to predict the properties of Mars' interior. These two events are described in [2], however subsequent work [12] has revealed that S1000a is a surface imapct at a known location. Using InSight_KKS21_GP, and the Taup Toolkit [30], Fig. S12 shows that a 50 km uncertainty in source depth would cause only a small difference ($\sim$ 3 s) in the predicted SS-SKS travel time for S1000a, while the difference in the SKS-PP time would be $\sim$ 8 s. An 8 s difference in travel time corresponds to less than 2% of the time that SKS spends in the core. Thus, while knowing that S1000a is a surface event is useful, it has not made a big impact on our assessment of the properties of Mars' core.

In contrast, the impact location for S1000a is 9.9° further from the initial Mars Quake Service estimate, though well within the uncertainty bounds published. The difference between the observed and estimated locations could have caused considerable difference to the expected arrival time.

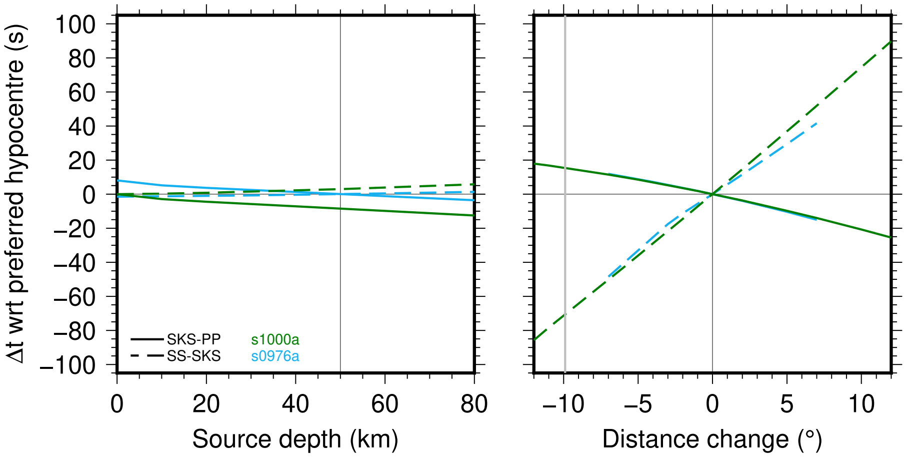

Figure S12: Impact on differential travel times of changing hypocentre. Predictions are made using the InSight_KKS21_GP model with and displayed relative to the preferred hypocentres for S0976a (distance used 146 degree, depth 50 km) and S0976a (distance used 125.9°, depth 0 km); these preferred hypocenters are indicated by black vertical lines. The grey vertical line indicates the epicentral distance of S1000a as estimated in [2].

3.3 Ray theoretical predictions: changing only Mars' core

In the InSight_KKS21_GP model, the core of Mars is assumed to be composed of an alloy in the Fe-FeS system, and no core-transiting eaves had been detected. Thus the seismic properties of the core in that model are not as well constrained as the elastic properties of the mantle. We can therefore use the crust and mantle of InSight_KKS21_GP, and adjust both the CMB velocity and core velocity gradient to attempt to fit the differential travel times.

Changing the CMB velocity is done by increasing all velocities across the core by a fixed value (from -0.6 km/s to 0.4 km/s) of the original. Changing the velocity gradient is achieved by applying no velocity change at the CMB, linearly increasing the velocity increase with depth in the core, so that the maximium velocity change is at the centre of Mars. CMB velocities gradients were varied so that the velocity gradient at the top of the core ranged between 0.2926 m/s/km to 0.9474 m/s/km. Core velocity models tested are shown in Figure S13. All models tested produced SKS-PP and SS-SKS differential travel times for s0976a. Of the two hundred and thirty one models tested, eighteen the with lowest velocities and lowest velocity gradients did not predict differential travel times for s1000a.

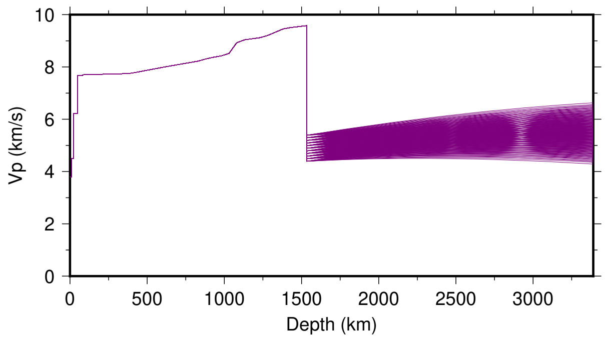

Figure S13: Models with a range of core velocities and core velocity gradients. The crust and mantle of every model is that of InSight_KKS21_GP, only the velocity of the core is changed.

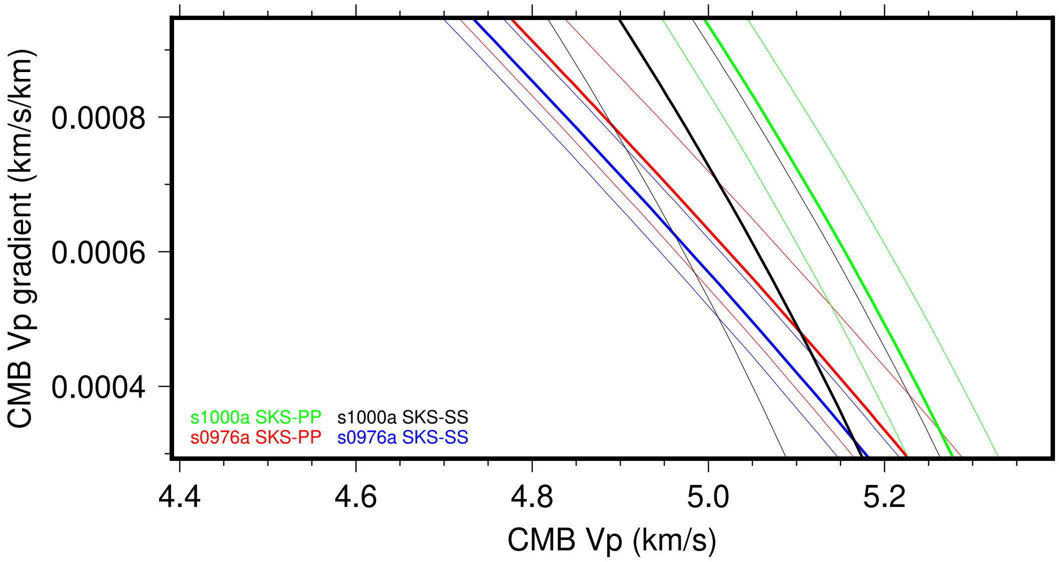

Figure S14: Changing the core's velocity at the CMB and the velocity gradient at the CMB away from that of InSight_KKS21_GP changes predicted SKS differential travel times. Thick coloured lines correspond to the CMB velocities and gradients which match the measured times; thin lines correspond to the measurements ± one st. dev. The CMB velocity of InSight_KKS21_GP is 4.991 km/s and the gradient at the CMB is 0.00062 km/s/km.

3.4 Waveform modelling of relative amplitudes

We used Instaseis [31] to create synthetic seismograms for events S0976a and S1000a. In this approach, waveforms are retrieved from a database of pre-computed Green's functions that were calculated using the AxiSEM software, a spectral element based solver of the elastic wave equation [32]. For this modeling, we used the database calculated for the Mars interior model InSight_KKS21_GP [10, 13, 7], which is publicly accessible through the ETH Zurich Green's function repository (`http://instaseis.ethz.ch/marssynthetics/`). For each event we calculated synthetic waveforms for a Mw 4 source and fixed the epicentral distances at 146.3° and 125.9° for events S0976a and S1000a, respectively. The event depths were assumed to be 20 km for S0976a and 0 km (i.e., a surface source) for S1000a. To assess the uncertainty in SKS amplitudes, as well as SKS/PP and SKS/SS amplitude ratios, due to radiation pattern effects, we calculated a suite of synthetic seismograms for 1000 randomly oriented double couple sources and stacked the resulting envelopes (Figures S15 and S16).

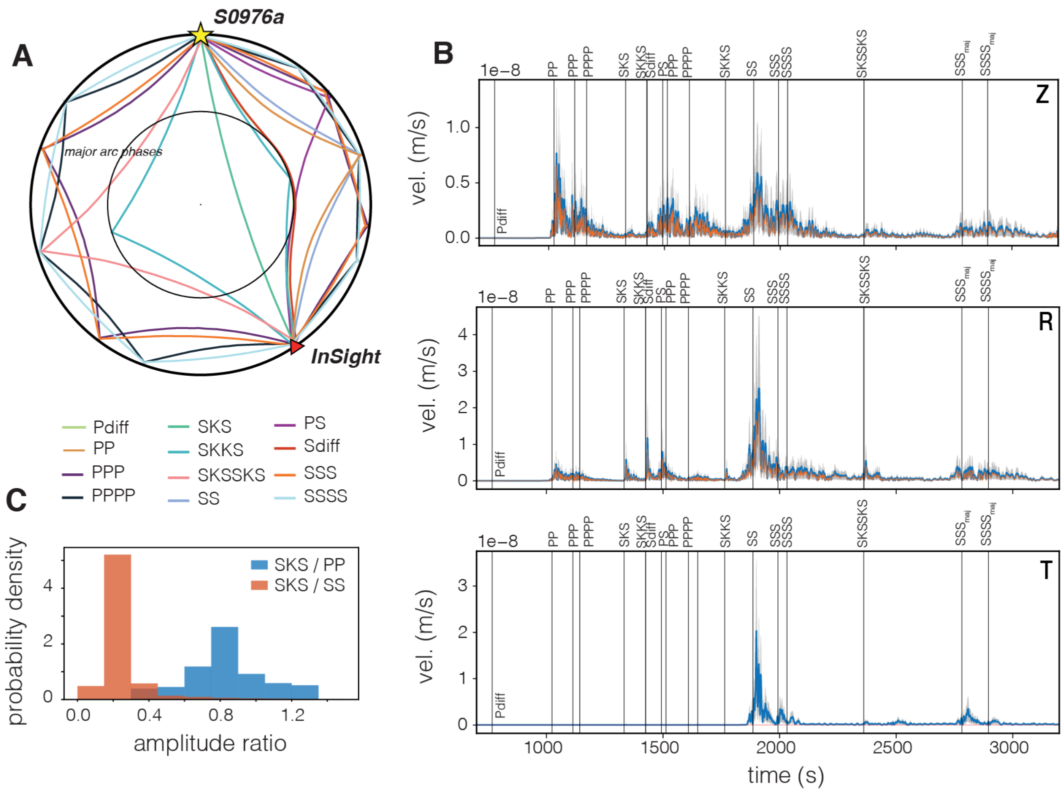

Figure S15: Synthetic waveform modeling of S0976a. (A) Raypath diagram of seismic phases that could be observed at InSight from S0976a. (B) 3-component synthetic envelope stacks. The blue line indicates the mean of the envelope stack, and the grey shaded region represents ± 1 standard deviation from the mean. The synthetic envelope for an isotropic moment tensor is shown in orange. (C) Histograms of the distribution of SKS/PP and SKS/SS amplitude ratios from the set of synthetics. Amplitudes of SKS and SS are measured on the radial component and PP is measured on the vertical component.

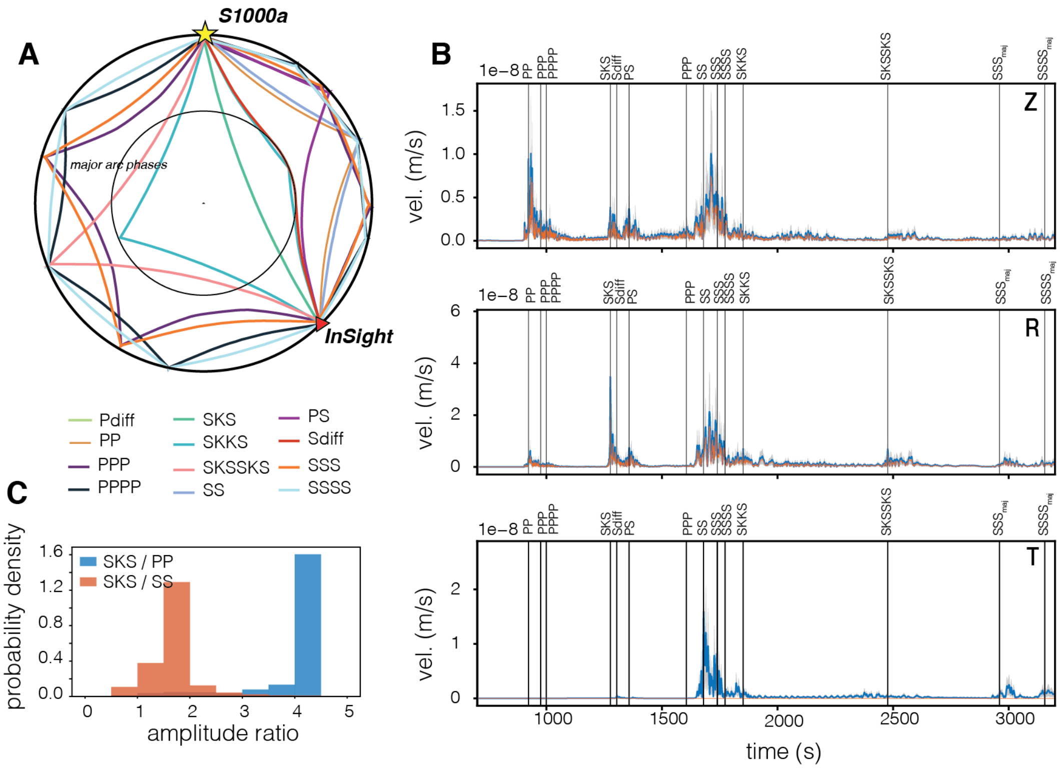

Figure S16: As Figure S15, but for S1000a.

4 Inversions

We employ two different inversion methods to assess the properties of Mars' interior. This reduces the risk of over-interpreting aspects of our inversion results which are associated with the approach taken instead of Mars itself. The approaches use the same core data (Table 1 in the main paper) and the same core parameterisation (the equation of state of a well mixed material), but they use slightly different mantle-sensitive data, and describe the mantle and crust in different ways. We also describe the methods used to interpret our results in terms of core composition. The following subsections contain details of the approaches taken.

4.1 Geodynamical inversions

4.1.1 Inversion parameterisation and methods

Following the approach described in [33, 34], we used a geodynamically-constrained inversion approach in which the models are parameterized in terms of quantities that influence the thermo-chemical evolution of the planet, accounting for 4.5 Gyr of planetary evolution. The resulting present-day thermo-chemical state of the planet is used to compute the density, and P- and S- waves seismic velocity profiles of Mars assuming compositional, mineralogical, and thermodynamic models for the different planetary envelopes. The latter consist of: (1) a convecting liquid iron-rich core; (2) an adiabatic convecting silicate mantle; and (3) a time-evolving stagnant lithospheric lid, which includes a crust enriched in heat-producing elements with respect to the underlying mantle. The thermo-chemical evolution is computed following the approach in [35] and references therein.

In the frame of this parameterization we invert for the following quantities: (i) the mantle rheology (i.e., its reference viscosity, η_0, effective activation energy, $E*$, and activation volume, $V*$), (ii) the planet's initial thermal state (i.e., the core mantle boundary T_{c0}, and the uppermost mantle temperature, T_{m0}), (iii) the crustal enrichment, $\mathbf{\Lambda}$ (i.e., the ratio of heat-producing elements content in the crust relative to that of the primitive mantle), (iv) the core size, R_c, and its density structure. The latter is parameterized in terms of a third-order Birch-Murnaghan Equation of State (EoS) for which we invert for the core density, ρ_{CMB}, and isentropic bulk modulus, Ks_{CMB}, at the Core-Mantle Boundary (CMB) pressure, together with and its pressure derivative Ks$'$.

Description	Value/Range
Crustal enrichment factor, $\mathbf{\Lambda}$	5-20
Initial uppermost mantle temperature, T_{m0}	1700 - 2000 K
Initial core-mantle boundary temperature, T_{c0}	$T_{c0} - T_{m0}$ = 300 - 600 K
Effective mantle activation energy, $E*$	60 - 500 kJ/mol
Reference mantle viscosity, η_0	$10^{20} - 10^{22.5}$ Pa s
Mantle activation volume, $V*$	0 - 10 cm^3/mol
Core radius, Rc	1500 - 2000 km
Isentropic bulk modulus, Ks_{CMB}	120 - 200 GPa
Isentropic bulk modulus pressure derivative, Ks'	3.5 - 7
Vs in the upper crust (layer 1)	1.0 - 3.0 km/s
Vs in the mid-crust (layer 2)	2.0 - 3.5 km/s
Vs in the lower crust (layer 3)	3.9 - 4.4 km/s
Vp/Vs in the entire crust	1.7 - 1.9
Source epicentral distance	0 - 180° (except for S1000a)
Source depth	5 - 200 km (except for S1000a)

Table S3: List of the inverted parameters and the corresponding prior bounds considered.

While the crustal thickness is an inversion output (the crust progressively forms by mantle melt extraction at shallow pressures) its complex seismic structure is decoupled from temperature, and is directly inverted for, using 3 crustal layers. In the lithosphere and in the mantle below the crust P- and S-wave seismic velocities and density are computed from the obtained thermo-chemical state at the present-day. The

computation of density and seismic velocities from thermal profiles are performed using a thermodynamic model via the Perple_X Gibbs free energy minimization software [36], using the database of [37]. In the mantle, the composition of [38] (hereafter termed EH45) and of [39] (hereafter termed YM20) are used on separate sets of inversions. These two compositions were chosen because they can yield density structures compatible with Mars' Moment of Inertia (MoI) factor, and with receiver function estimations and gravity and topography inversions [13]. The core density ρ_{CMB} is adjusted to match the mean mass of Mars, $M = 6.417 \times 10^{23} \pm 2.981 \times 10^{19}$ kg [40]. In the core, the P-wave velocities are then computed from the corresponding values of Ks and the density obtained from the Birch-Murnaghan EoS along the radial profile. The average crustal density is adjusted within bounds compatible with receiver function estimations and gravity and topography considerations [13]. Models for which crustal density cannot satisfy these aforementioned constraints together with a normalized MoI factor of 0.3634 ± 0.00006 [40] were rejected. We verified that the large fraction of the best retained output models are compatible with degree-two Love number (k2) estimates [40].

As in [33, 34], we also used an inversion approach in which the models are parameterized in terms of seismic velocities as a function of depth. These models also have three layers in the crust, and the seismic velocities in the mantle and core are constructed using Bézier curves. This more classical inversion approach serves to test the resolution power of the seismic data alone. For both parameterizations, the crustal thickness is allowed to randomly vary between 39 and 72 km, which corresponds to the estimation of the average thickness of the Martian crust based on receiver functions [13]. The prior model parameter information described above is summarized in Table S3 .

The body wave arrival times are computed using the TauP software [30]. To solve the inverse problem, we use a Bayesian method based on a Markov chain Monte Carlo approach [41]. The differential arrival times data set of [34] is employed, except for S0976a and S1000a, for which the differential arrival times in Table 1 of the main paper are used. In total, 19 marsquakes are considered. The epicentral distance and the depth of the events are randomly sampled within ranges of 0-180° and 5-200 km, respectively, except for S1000a for which the source location is fixed at the surface of Mars at an epicentral distance of 125.9° [12].

4.1.2 Inversion results

Our inversion results show that the data are compatible with a core radius of 1790±43 km and 1757±37 km considering the EH45 and YM20 compositions, respectively (FigureS17 a1-a2). Due to the trade-off between the seismic velocities in the mantle and the core radius, the Vp and Vs seismic velocities in the mantle are larger for YM20 compared to EH45 (Figure S18a-b). Using the classical parameterization, the mean core radius is equal to 1798±45 km (Figure S19). The YM20 composition implies a denser core (6443±156 kg/m^3) compared to the value obtained with the EH45 composition (6290±172 kg/m^3) (Figure S17b1-b2). The Ks_{CMB} values are nearly similar in both cases (Figure S17d1-d2), with a mean value of 153±11 GPa for EH45 and 146±10 GPa for YM20. As illustrated by the similarity in the prior and posterior marginal distributions, the Ks' value is not constrained by our data set. The datafit in Figure 4 shows that the sampled models are able to explain the differential time tPP-tSKS of S0976a and S1000a within the uncertainty bars, for both compositions. However, the YM20 models have more difficulty to fit the tSS-tPP differential time of S1000a (Figure S20b2). The models with this composition that satisfy the data (within the one sigma uncertainties, Figure S17b2) exhibit a present-day potential mantle temperature Tp of 1635±15 K together with a lithosphere thickness in excess of 700 km (including the upper thermal boundary layer), while those that are unable to satisfy the tSS-tPP differential time of S1000a are associated with a considerably colder mantle (Tp=1510±35 K) and lithospheres that are 200 km to 300 km thinner. Even though the aforementioned YM20 models composition can satisfy the data, their extremely thick lithosphere would be difficult to reconcile with Mars' estimates of elastic thickness [42]. EH45 models have a smaller heat-producing element content and consequently exhibit more reasonable lithospheric thicknesses (550±50 km including the upper thermal boundary layer for the best 600 models) at the present-day.

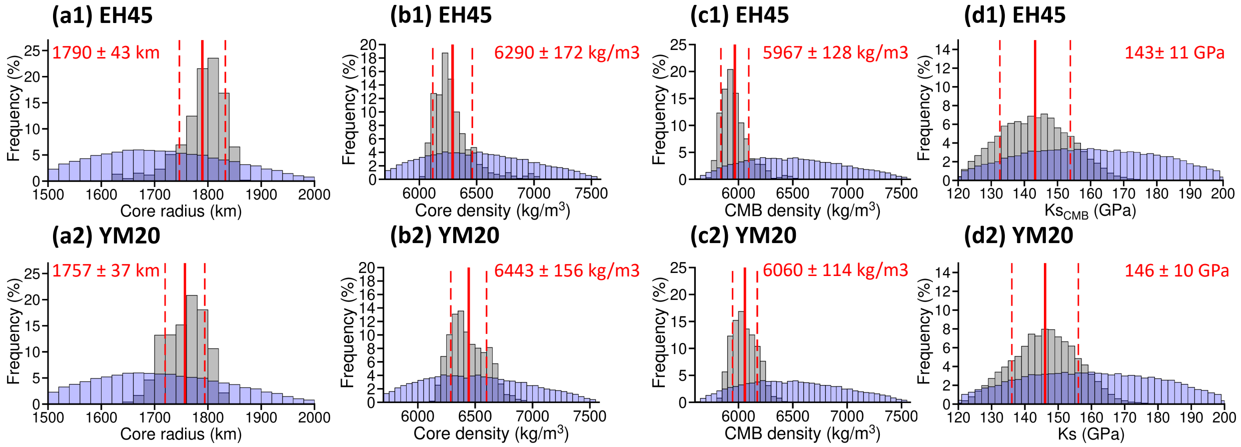

Figure S17: Marginal distributions of the core radius (a1, a2), the core density (b1, b2), the density at the CMB (c1, c2), and Ks_{CMB} (d1, d2), considering the compositions of [38] and [39] (top and bottom rows, respectively). The blue and gray histograms correspond to the *a priori* and *a posteriori* distributions, respectively. The mean values and their one sigma uncertainties are shown with red lines.

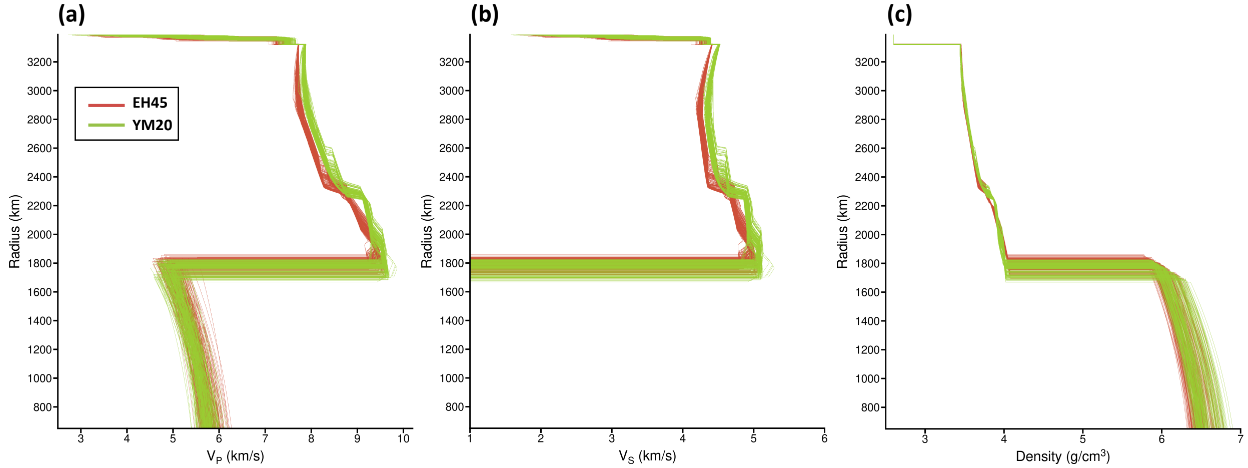

Figure S18: Inversion results for Vp (a), Vs (b) and density (c) profiles. A random subset of 200 models selected from the ensemble solution is shown. Results considering the mineralogical compositions of [38] and [39] are displayed in red and green, respectively.

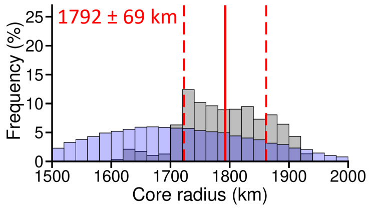

Figure S19: Marginal distributions of the core radius obtained considering the classical parameterization. The blue and gray histograms correspond to the *a priori* and *a posteriori* distributions, respectively. The mean values and their one sigma uncertainties are shown with red lines.

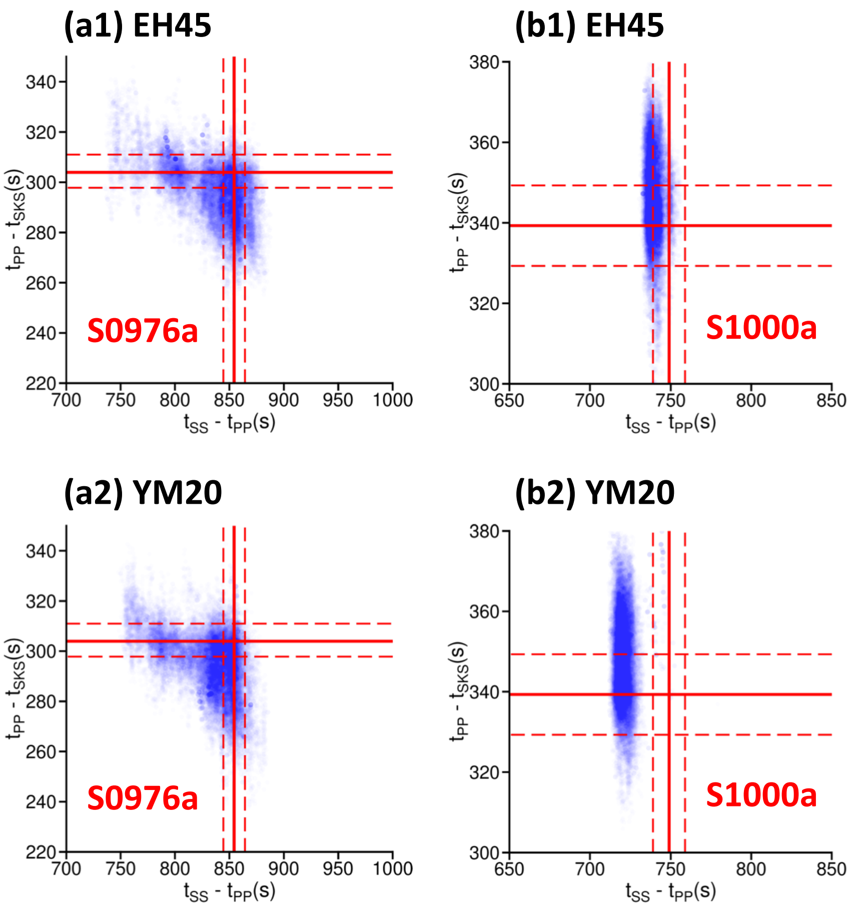

Figure S20: Datafit of the differential arrival times tPP-tSKS as a function of tSS-tPP, for S0976a (a1, a2) and S1000a (b1, b2), considering the compositions of [38] and [39] (top and bottom rows, respectively). The differential arrival time measurements and their one sigma uncertainties are shown with red lines.

4.2 Geophysical inversion

4.2.1 Model parameterization

We consider a spherically symmetric model of Mars, divided intro three regions comprising crust, mantle, and core. The crust is divided into three layers that are described by variable S-wave velocity and thickness. Crustal S-wave velocities are assumed to increase as a function of depth, while density and P-wave velocity are scaled to S-wave velocity using variable but depth-independent scaling factors.

Seismic P- and S-wave velocity profiles in the sub-lithospheric mantle are computed using phase equilibria computations in the CaO-FeO-MgO-Al_2O_3-SiO_2-NaO_2 (CFMASNa) model chemical system. The stable mantle mineralogy and physical properties are computed employing Gibbs free-energy minimization and equation-of-state modelling [43] as a function of temperature, pressure, and composition, using the thermodynamic formulation and parameters described in [44, 37]. The lithospheric thermal structure is described by a conductive geothermal gradient between the crust-mantle interface (variable) and the bottom of the lithosphere (variable). The thermal structure of the mantle is assumed to be adiabatic and mantle adiabats (isentropes) are computed from the entropy of the lithology at the pressure and temperature of the bottom of the thermal lithosphere. The mantle pressure profile is obtained by integrating the vertical load from the surface pressure boundary condition.

We assume the core of Mars to be homogeneous, convecting, and well-mixed and rely on a third-order Birch-Murnaghan EoS to compute seismic properties (density and P-wave velocity). This EoS is param-

eterised using parameters at CMB conditions: bulk modulus (K_{0S}), the pressure derivative of the bulk modulus (K'_{0S}), and density ϱ_0. Prior information is described in detail in Table S4.

4.2.2 Inverse problem

To solve the inverse problem of determining seismic velocity profiles and marsquake epicentral distances, we employ the probabilistic approach of [45], with a solution given by

$$\sigma(\mathbf{m}) = kf(\mathbf{m})\mathcal{L}(\mathbf{m}), \tag{1}$$

where k is a normalization constant, $f(\mathbf{m})$ is the prior model parameter probability distribution, $\mathcal{L}(\mathbf{m})$ is the likelihood function, which is a measure of the similarity between the observed data and the predictions from model $\mathbf{m}$, and $\sigma(\mathbf{m})$ is the posterior probability distribution. The particular form of $\mathcal{L}(\mathbf{m})$ is determined by the observations, their uncertainties, and how these are employed to model data noise.

Assuming that data noise is uncorrelated and described by a Laplace distribution (L_1-norm), the likelihood function takes the form

$$\mathcal{L}(\mathbf{m}) \propto \prod_{\nu} \exp\left(-\Phi^{\nu}\right), \tag{2}$$

where Φ is the misfit function and ν represents considered data set. Following the work of [14], our data set consists of receiver functions (RF), differential travel times, mean moment of inertia (I/MR^2) and mean density ($\overline{\rho}$), with the additional differential travel times of the two new events: S0976a and S1000a. The general expression for the misfit is

$$\Phi^{\nu} = \frac{1}{N}\sum_{j}^{N} \frac{|\mathbf{d}^{\nu}_{\mathrm{obs_j}} - \mathbf{d}^{\nu}_{\mathrm{cal_j}}|}{\sigma^{\nu}_{j}}. \tag{3}$$

• In the case of the stacked RF, $\mathbf{d}_{\mathrm{obs}}$ and $\mathbf{d}_{\mathrm{cal}}$ denote the vectors of observed and synthetic amplitudes of the stacked Ps RF, and σ the data uncertainty, with N expressing the total number of points within the misfit window. The uncertainty σ is set to 50% of the mean absolute Ps RF amplitude within the misfit window 0–8.7 s, covering the three main peaks of the RF signal

• For the differential travel times, $\mathbf{d}_{\mathrm{obs}}$ and $\mathbf{d}_{\mathrm{cal}}$ denote the vectors of observed and synthetic differential travel times, with N expressing the total number of differential travel times, which comprises all possible combinations of phases with respect to P and S: T_S–T_P, T_{pP}–T_P, T_{PP}–T_P,..., T_{SS}–T_{PP}, T_{SS}–T_S, T_{SSS}–T_S, T_{ScS}–T_S, and T_{SKS}–T_{SS}.

Finally, to sample the posterior distribution (Eq. 1), we employ the Metropolis sampling algorithm [45]. This algorithm ensures that models that fit the data and are simultaneously consistent with prior information are sampled more frequently.

Table S4: Overview of model parameters, their quantity, prior model ranges and probability distributions employed in the geophysical parameterization

Description	Parameter	Quantity	Value/range	Distribution
**Crust**				
S-wave velocity	V_S^i	3	1.5–4.2 km/s	uniform[1]
P-wave velocity	V_P^i	-	$\alpha \cdot V_S^i$	uniform[2]
V_P/V_S scaling	α	1	1.65–1.85	uniform
Moho depth	Z_c	1	20–60 km	uniform
Layers thickness	ΔZ_i	3	1–50 km	uniform
**Mantle**				
Lithospheric temperature	T_{lit}	1	1273–1873 K	uniform
Lithospheric depth	Z_{lit}	1	100–600 km	uniform
Mantle composition	X_m	1	[46]<br>[38]<br>[47]	fixed
**Core**				
Core radius	R_{cmb}	1	1500–2300 Km	uniform
Bulk modulus	K0	1	120–200	uniform
Bulk modulus pressure derivative	$K0'$	1	3.5–8	uniform
Density	$\rho 0$	1	4.5–7.0 g/cm^3	uniform
**Marsquakes**				
Epicentral distance (S1000a)	Δ	1	125.9	fixed
Epicentral distance (all other events)	Δ	16	0°–180°	uniform
Depth (S1000a)	h	1	0	fixed
Depth (all other events)	h	16	10–100 km	[14]

[1] V_S increases with depth.

[2] V_P increases with depth.

4.3 Full seismic models

In Fig. S21 we show full density and P-wave velocity profiles derived using the *geophysical* and *geodynamical* inversion methods.

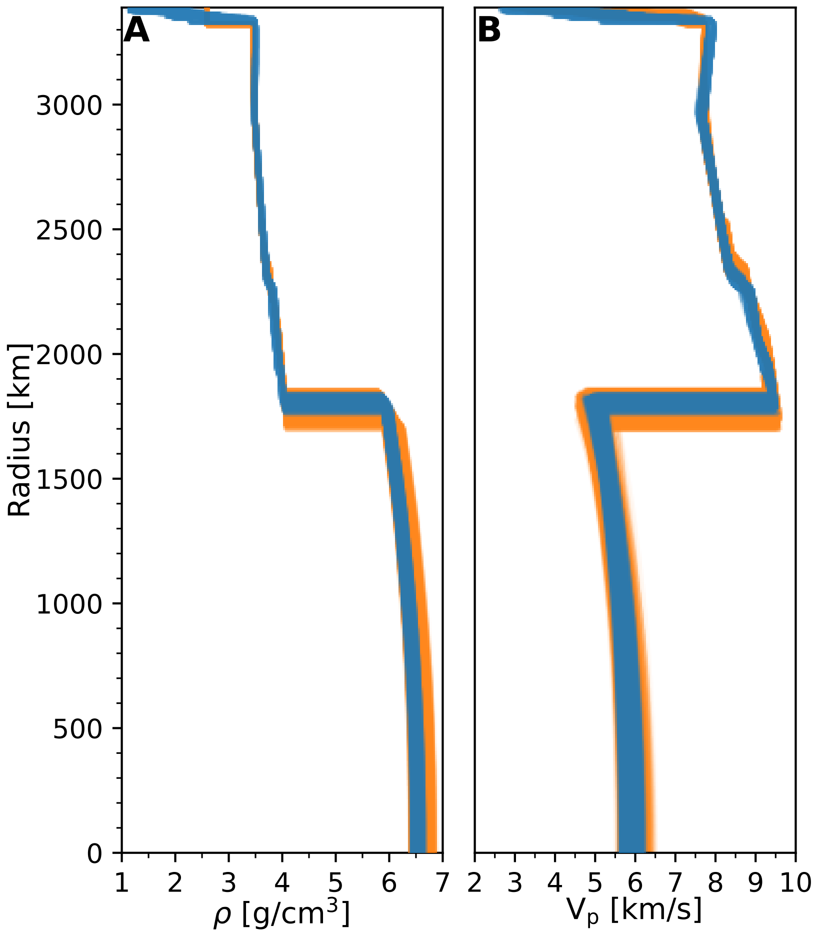

Figure S21: Inverted density and seismic velocity models for Mars. *Geophysical* inversion results are shown in blue, *Geodynamical* results are shown in orange. Panel (A) shows density and panel (B) shows P-wave velocity.

4.4 Equation of state for the liquid core

We model the core under the assumption that it is made by an iron alloy containing sulfur (S), oxygen (O), carbon (C), and hydrogen (H) as light elements (given in decreasing order of occurrence). All four light elements are siderophile at core forming conditions [e.g. 48] and abundant enough to significantly affect the density and sound velocity of the core.

We describe the equation of state of the liquid Fe-S-O-C-H by an asymmetric Margules mixing model that uses Fe, FeS, FeO, Fe_3C, and FeH as end-members. The volume of the solution writes as

$$V(\{\chi_i\}, p, T) = V_{\text{ideal}}(\{\chi_i\}, p, T) + V_{\text{ex}}(\{\chi_i\}, p) \;, \tag{4}$$

where

$$V_{\text{ideal}}(\{\chi_i\}, p, T) = \sum_{i=\{\text{Fe},\text{FeS},\text{FeO},\text{Fe}_3\text{C},\text{FeH}\}} \chi_i V_i(p, T) \;,$$

	T_0 K	V_0 cm^3/mol	C_P J/K/mol	α $10^5/K$	K_T GPa	K'_T	γ	δ_T	W_{Fe-X} cm^3/mol	W_{X-Fe} cm^3/mol	B_0 GPa	B'_0
FeS	1650	24.4±0.3	62.5	11.8	12.0±-0.8	6.9±0.3	0.62±0.04	0.4±-0.5	-9.9±-1.4	-3.54±-0.4	3.02±-0.3	2.6±-0.4
Fe_3C	1723	26.5±0.1	215.	14.8±5	57.5±13	15.±3	1.34±0.4	9.1±4.0	-	-	-	-

Table S5: Equation of state parameters for the Anderson-Grünisen equation [e.g. 49] and Margules coefficients. κ=1.4 and X stands for FeS or Fe_3C.

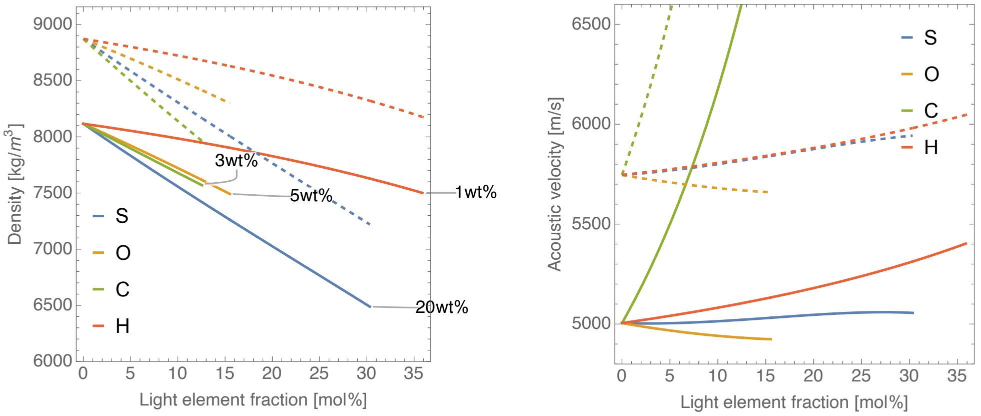

Figure S22: Effect of light element concentration on density (right) and acoustic velocity (left) at Mars' core-mantle boundary conditions (full lines) (p = 20 GPa, T = 2000 K) and core center conditions (dashed lines) (p = 40 GPa, T = 2000 K)

is the ideal contribution to the volume and

$$V_{\text{ex}}(\{\chi_i\}, p) = \chi_{\text{Fe}}\chi_{\text{FeO}}\left(\chi_{\text{FeO}}W_{\text{Fe}-\text{FeO}} + \chi_{\text{Fe}}W_{\text{FeO}-\text{Fe}}\right) + \chi_{\text{Fe}}\chi_{\text{FeS}}\left(\chi_{\text{FeS}}W_{\text{Fe}-\text{FeS}}(p) + \chi_{\text{Fe}}W_{\text{FeS}-\text{Fe}}(p)\right)$$

the excessive contribution. The χ_i and V_i are the molar fractions and molar volumes of Fe, FeS, FeO, Fe_3C, and FeH, and the W_{i-j} are the Margules coefficients for Fe-FeO [49] and Fe-FeS [50].

Molar volumes of the end-members are computed on the basis of their equation of state (EoS). We use [51] for Fe, [52] for FeO, and [53] for FeH. The EoS for liquid FeS and Margules coefficients for Fe − FeS are deduced from elastic data used in [50] and high pressure density (up to 43GPa) [54] and acoustic velocity data (up to 50GPa) [55, 56]. Finally, the EoS for liquid Fe_3C is deduced from density and acoustic data [57, 58, 59] acquired at pressures as high as 53 GPa. The equation of state parameters and Margules parameters for FeS and Fe_3C are given in Table S5. From the EoS of the end-members and Eq. [4] all relevant thermodynamic quantities required to compute adiabatic gradient and acoustic velocity in the core are obtained by applying classical thermodynamic relations.

The addition of light elements to Fe decreases its density but the effect is more complex for acoustic velocity (see Fig. S22). In particular, the pressure-dependent non-ideal mixing of liquid FeS into liquid Fe results in an initial increasing velocity that reaches a maximum and can later decreases below the velocity of liquid Fe (depending on pressure) for large fractions of S. We note however that at pressure and temperature condition of the Martian core mantle boundary, the variation is rather small. The effect is larger for O, H and C. The velocity decreases with O and it increases with H and substantially with C.

4.5 Core composition inference

In order to deduce the composition of the core that best matches the seismologically inferred equation of state parameters ρ_0, $K_{S,0}$, and $K'_{S,0}$ of the isentropic BM3 equation, we first seek the weight fractions of S and H that minimize the following expression:

$$\left(\rho_0 - \rho(x_S, x_H, p_{cmb}, T_{cmb})\right)^2, \tag{5}$$

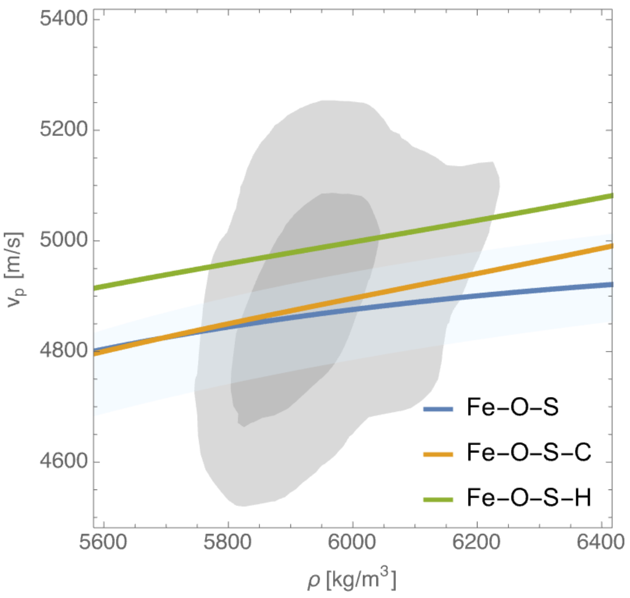

Figure S23: Density-velocity relation for Fe-O-S, Fe-O-S-C, and Fe-O-S-C-1wt%H at (20 GPa, 2000 K). The blue shaded area is representative for the effect of pressure and temperature variation on the Fe-O-S alloy. The gray areas show 50% and 90% confidence regions for the density-velocity probability density function for the EH45 mantle composition using the *geodynamical* approach with SKS data.

where ρ is the density of the liquid Fe-S-O-C-H alloy at *cmb* pressure and temperature and x_S and x_H are the weight fractions of S and H.

The amount of O in the core is not a free parameter, as it depends on the conditions the core has formed, in particular on the oxygen fugacity and on the amount of S. Here we use a parameterization that relates the O fraction in the core to the amount of S and fraction of FeO in the mantle. The relation has been deduced from a multi-stage core formation model that takes into account the chemical interaction of the magma ocean and core forming metal [60]. The amount of C is also not a free parameter, it is mainly limited by the amount of S in the core. In our study we set to it to its solubility limit in the Fe-S alloy [61].

A large number of S-H weight fraction pairs that agree with ρ_0 have a $K_{S,0}$ that differs significantly from that inferred from seismic data. In other words, an appreciable subset of the equation of state parameters inferred from seismic data is not compatible with elastic properties of the liquid Fe-O-S-C-H alloy of our study (see Fig. S23). For this reason, in a second step, we only retain the subset of seismic models that agree with ρ and v_p of the alloy within respectively 0.1% and 2% (see main text).

4.5.1 Extended analysis of seismic models of inversions

Figure S24 shows the results of the compositional interpretation of the *geodynamical* models, with the sulfur content of each model compared to the hydrogen content. There is a strong anti-correlation evident - cores with higher sulfur fractions have lower hydrogen fractions.

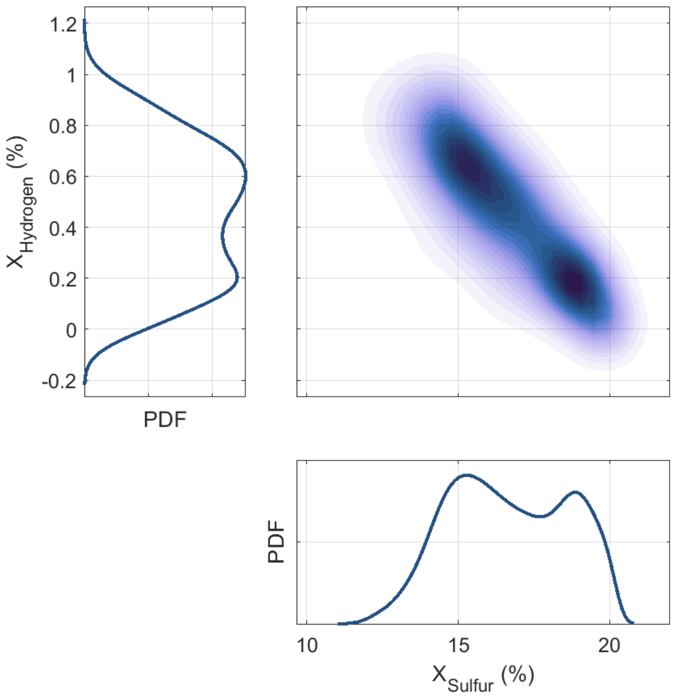

Figure S24: Fraction of sulfur (horizontal axis and PDF) and hydrogen (vertical axis and PDF) in Mars' inferred from the results of the *geodynamical* inversion.

Bibliography

[1] Banerdt WB, et al. (2020) Initial results from the InSight mission on Mars. *Nat. Geosci.* 13(3):183–189.

[2] Horleston AC, et al. (2022) The far side of Mars: two distant marsquakes detected by InSight. *The Seismic Record* 2(2):88–99.

[3] Li J, et al. (2022) Constraints on the Martian crust away from the InSight landing site. *Nat. Commun.* 13:7950.

[4] Scholz JR, et al. (2020) Detection, analysis, and removal of glitches from InSight's seismic data from Mars. *Earth Space Sci.* 7(11):e2020EA001317.

[5] Kim D, et al. (2021) Potential Pitfalls in the Analysis and Structural Interpretation of Seismic Data from the Mars InSight Mission. *Bull. Seismol. Soc. Am.* 111(6):2982–3002.

[6] Park J, Vernon FL, III, Lindberg CR (1987) Frequency dependent polarization analysis of high-frequency seismograms. *J. Geophys. Res.* 92(B12):12664–12674.

[7] Stähler SC, et al. (2021) Seismic detection of the martian core. *Science* 373(6553):443–448.

[8] Kim D, et al. (2022) Surface waves and crustal structure on Mars. *Science* 378(6618):417–421.

[9] Montalbetti JF, Kanasewich ER (1970) Enhancement of teleseismic body phases with a polarization filter. *Geophys. J. Int.* 21(2):119–129.

[10] Khan A, et al. (2021) Upper mantle structure of Mars from InSight seismic data. *Science* 373(6553):434–438.

[11] Huang Q, et al. (2022) Seismic detection of a deep mantle discontinuity within Mars by InSight. *Proc. Natl. Acad. Sci. USA* 119(42):e2204474119.

[12] Posiolova LV, et al. (2022) Largest recent impact craters on Mars: Orbital imaging and surface seismic co-investigation. *Science* 378(6618):412–417.

[13] Knapmeyer-Endrun B, et al. (2021) Thickness and structure of the Martian crust from InSight seismic data. *Science* 373(6553):438–443.

[14] Durán C, et al. (2022) Seismology on Mars: An analysis of direct, reflected, and converted seismic body waves with implications for interior structure. *Phys. Earth Planet. Inter.* 325:106851.

[15] InSight Marsquake Service (2022) Mars Seismic Catalogue, InSight Mission; V11 2022-07-01.

[16] Durán C, et al. (2022) Observation of a core-diffracted P-wave from a farside impact with implications for the lower-mantle structure of Mars. *Geophys. Res. Lett.* 49:e2022GL100887.

[17] Schimmel M (1999) Phase cross-correlations: Design, comparisons, and applications. *Bull. Seismol. Soc. Am.* 89(5):1366–1378.

[18] Medeiros WE, Schimmel M, do Nascimento AF (2015) How much averaging is necessary to cancel out cross-terms in noise correlation studies? *Geophys. J. Int.* 203(2):1096–1100.

[19] Schimmel M, Stutzmann E, Ventosa S (2018) Low-frequency ambient noise autocorrelations: Waveforms and normal modes. *Seismol. Res. Lett.* 89(4):1488–1496.

[20] Ventosa S, Schimmel M, Stutzmann E (2019) Towards the processing of large data volumes with phase cross-correlation. *Seismol. Res. Lett.* 90(4):1663–1669.

[21] Schimmel M, et al. (2021) Seismic noise autocorrelations on Mars. *Earth Space Sci.* 8(6):e2021EA001755.

[22] Haney MM, et al. (2020) Co-eruptive tremor from Bogoslof volcano: seismic wavefield composition at regional distances. *Bull. Volcanol.* 82(2):1–14.

[23] Charalambous C, et al. (2021) A comodulation analysis of atmospheric energy injection into the ground motion at InSight, Mars. *J. Geophys. Res. Planets* 126(4):e2020JE006538.

[24] Clinton JF, et al. (2021) The Marsquake catalogue from InSight, sols 0—478. *Phys. Earth Planet. Inter.* 310:106595.

[25] Ceylan S, et al. (2021) Companion guide to the marsquake catalog from InSight, Sols 0–478: Data content and non-seismic events. *Phys. Earth Planet. Inter.* 310:106597.

[26] Ceylan S, et al. (2022) The marsquake catalogue from InSight, sols 0–1011. *Phys. Earth Planet. Inter.* 333:106943.

[27] Banfield D, et al. (2019) InSight auxiliary payload sensor suite (APSS). *Space Sci. Rev.* 215(1):1–33.

[28] Banfield D, et al. (2020) The atmosphere of Mars as observed by InSight. *Nat. Geosci.* 13(3):190–198.

[29] Dahmen NL, et al. (2021) Resonances and lander modes observed by InSight on Mars (1–9 Hz). *Bull. Seismol. Soc. Am.* 111(6):2924–2950.

[30] Crotwell HP, Owens TJ, Ritsema J (1999) The TauP Toolkit: Flexible Seismic Travel-time and Ray-path Utilities. *Seismol. Res. Lett.* 70(2):154–160.

[31] van Driel M, Krischer L, Stähler S, Hosseini K, Nissen-Meyer T (2015) Instaseis: Instant global seismograms based on a broadband waveform database. *Solid Earth* 6(2):701–717.

[32] Nissen-Meyer T, et al. (2014) AxiSEM: broadband 3-D seismic wavefields in axisymmetric media. *Solid Earth* 5(1):425–445.

[33] Drilleau M, Samuel H, Rivoldini A, Panning M, Lognonné P (2021) Bayesian inversion of the Martian structure using geodynamic constraints. *Geophys. J. Int.* 226(3):1615–1644.

[34] Drilleau M, et al. (2022) Marsquake locations and 1-D seismic models for Mars from InSight data. *J. Geophys. Res. Planets* 127(9):e2021JE007067.

[35] Samuel H, Lognonné P, Panning M, Lainey V (2019) The rheology and thermal history of Mars revealed by the orbital evolution of Phobos. *Nature* 569(7757):523–527.

[36] Connolly J (2005) Computation of phase equilibria by linear programming: A tool for geodynamic modeling and its application to subduction zone decarbonation. *Earth Planet. Sci. Lett* 236(1):524–541.

[37] Stixrude L, Lithgow-Bertelloni C (2011) Thermodynamics of mantle minerals - II. Phase equilibria. *Geophys. J. Int.* 184(3):1180–1213.

[38] Sanloup C, Jambon A, Gillet P (1999) A simple chondritic model of Mars. *Phys. Earth Planet. Inter.* 112(1):43–54.

[39] Yoshizaki T, McDonough WF (2020) The composition of Mars. *Geochim. Cosmochim. Acta* 273:137–162.

[40] Konopliv AS, et al. (2020) Detection of the chandler wobble of Mars from orbiting spacecraft. *Geophys. Res. Lett.* 47(21):e2020GL090568.

[41] Tarantola A (2005) *Inverse problem theory and methods for model parameter estimation.* (SIAM).

[42] Grott M, Breuer D (2008) The evolution of the martian elastic lithosphere and implications for crustal and mantle rheology. *Icarus* 193(2):503–515.

[43] Connolly JAD (2009) The geodynamic equation of state: What and how. *Geochem. Geophys. Geosyst.* 10(10):Q10014.

[44] Stixrude L, Lithgow-Bertelloni C (2005) Thermodynamics of mantle minerals - I. Physical properties. *Geophys. J. Int.* 162(2):610–632.

[45] Mosegaard K, Tarantola A (1995) Monte carlo sampling of solutions to inverse problems. *J. Geophys. Res.* 100(B7):12431–12447.

[46] Lodders K, Fegley B (1997) An oxygen isotope model for the composition of Mars. *Icarus* 126(2):373–394.

[47] Taylor J, Teanby NA, Wookey J (2013) Estimates of seismic activity in the Cerberus Fossae region of Mars. *J. Geophys. Res. Planets* 118(12):2570–2581.

[48] Steenstra ES, van Westrenen W (2018) A synthesis of geochemical constraints on the inventory of light elements in the core of mars. *Icarus* 315:69–78.

[49] Komabayashi T (2014) Thermodynamics of melting relations in the system Fe-FeO at high pressure: Implications for oxygen in the Earth's core. *J. Geophys. Res.* 119(5):4164–4177.

[50] Xu F, et al. (2021) Thermal expansion of liquid Fe-S alloy at high pressure. *Earth Planet. Sci. Lett* 563:116884.

[51] Dorogokupets PI, Dymshits AM, Litasov KD, Sokolova TS (2017) Thermodynamics and Equations of State of Iron to 350 GPa and 6000 K. *Sci. Rep.* 7:41863.

[52] Morard G, et al. (2022) Structural and electronic transitions in liquid FeO under high pressure. *Journal of Geophysical Research: Solid Earth* 127(11):e2022JB025117.

[53] Tagawa S, Helffrich G, Hirose K, Ohishi Y (2022) High-Pressure Melting Curve of FeH: Implications for Eutectic Melting Between Fe and Non-Magnetic FeH. *J. Geophys. Res.* 127(6):e2022JB024365.

[54] Morard G, et al. (2013) The Earth's core composition from high pressure density measurements of liquid iron alloys. *Earth Planet. Sci. Lett* 373:169–178.

[55] Kawaguchi SI, et al. (2017) Sound velocity of liquid Fe-Ni-S at high pressure. *J. Geophys. Res.* 122(5):3624–3634.

[56] Nishida K, et al. (2020) Effect of sulfur on sound velocity of liquid iron under Martian core conditions. *Nat. Commun.* 11(1):1954.

[57] Terasaki H, et al. (2010) Density measurement of Fe3C liquid using X-ray absorption image up to 10 GPa and effect of light elements on compressibility of liquid iron. *J. Geophys. Res.* 115:B06207.

[58] Morard G, et al. (2017) Structure and density of Fe-C liquid alloys under high pressure. *J. Geophys. Res.* 122(10):7813–7823.

[59] Shimoyama Y, et al. (2013) Density of Fe-3.5C liquid at high pressure and temperature and the effect of carbon on the density of the molten iron. *Phys. Earth Planet. Inter.* 224:77–82.

[60] Gendre H, Badro J, Wehr N, Borensztajn S (2022) Martian core composition from experimental high-pressure metal-silicate phase equilibria. *Geochem. Perspect. Lett.* 21:42–46.

[61] Tsuno K, Grewal DS, Dasgupta R (2018) Core-mantle fractionation of carbon in Earth and Mars: The effects of sulfur. *Geochim. Cosmochim. Acta* 238:477–495.
